# CircRAD23B-208aa Promotes Gastric Cancer Progression by Activating the Unfolded Protein Response through PDIA5 SUMOylation

**DOI:** 10.34133/research.1357

**Published:** 2026-07-17

**Authors:** Yuli Chen, Jiahao Guo, Ziwei Li, Shaokun Yu, Xiao Ke, Qinnan Chen, Hao Wu, Ming Sun, Fengqi Nie, Xianghua Liu

**Affiliations:** ^1^ Department of Oncology, Suzhou Cancer Center Core Laboratory, Oncology Laboratory of Medical Science and Technology Innovation Center, The Affiliated Suzhou Hospital of Nanjing Medical University, Suzhou Municipal Hospital, Gusu School, Nanjing Medical University, Suzhou, Jiangsu Province, China.; ^2^ Department of Transfusion Medicine, Key Laboratory of Jiangxi Province for Transfusion Medicine, The First Affiliated Hospital, Jiangxi Medical College, Nanchang University, Nanchang, Jiangxi Province, China.; ^3^ Department of Oncology, The First Affiliated Hospital of Nanjing Medical University, Nanjing, Jiangsu Province, China.; ^4^ Gastric Cancer Center, The First Affiliated Hospital of Nanjing Medical University, Nanjing, Jiangsu Province, China.; ^5^ Institute for Gastric Cancer Research, Nanjing Medical University, Nanjing, Jiangsu, China.; ^6^ Department of Oncology, Second Affiliated Hospital, Nanjing Medical University, Nanjing, Jiangsu Province, China.; ^7^ Department of Biochemistry and Molecular Biology, School of Basic Medical Sciences, Nanjing Medical University, Nanjing, Jiangsu Province, China.

## Abstract

Gastric cancer (GC) remains a lethal malignancy with limited therapeutic options and poor prognosis. In this study, we employed integrated RNA sequencing and ribosome nascent-chain complex sequencing analyses to identify a coding circular RNA (circRNA), circRAD23B, which is markedly up-regulated in GC tissues. Its expression correlates strongly with advanced tumor stage, lymph node metastasis, and reduced overall survival. We further demonstrate that the splicing factor U2AF65 facilitates circRAD23B biogenesis through direct binding to intron 1 of the RAD23B pre-mRNA. Functionally, circRAD23B encodes a novel 208-amino acid protein via an internal ribosome entry site-dependent mechanism. This protein promotes GC proliferation, invasion, and lung metastasis in vivo. Mechanistically, circRAD23B-208aa recruits the E2 ligase UBC9 to catalyze SUMOylation of PDIA5 at lysine 25, thereby attenuating its ubiquitination and enhancing protein stability. Stabilized PDIA5 facilitates ATF6 activation by promoting its proteolytic processing, nuclear translocation, and transcriptional induction of key unfolded protein response (UPR) effectors—TXNRD1 and HERPUD1—thereby alleviating endoplasmic reticulum stress and promoting tumor survival. Our findings reveal circRAD23B-208aa as the first circRNA-encoded activator of the UPR pathway via posttranslational regulation of PDIA5, highlighting the therapeutic potential of targeting the SUMOylation-dependent PDIA5/ATF6 axis in GC.

## Introduction

According to the latest estimates from GLOBOCAN 2022, gastric cancer (GC) continues to pose a important global health burden, ranking among the top malignancies in both incidence and mortality within the gastrointestinal tract [1]. Although recent advances in targeted therapies (e.g., anti-HER2 agents), immune checkpoint inhibitors, and precision surgical techniques have modestly improved patient outcomes, the high heterogeneity of GC, challenges in early diagnosis, and the emergence of chemoresistance remain major clinical hurdles [2,3]. In this context, a deeper understanding of the molecular mechanisms underlying gastric carcinogenesis and progression is essential to identify novel diagnostic biomarkers and therapeutic targets that may overcome current treatment limitations.

Circular RNAs (circRNAs) represent a class of endogenous, covalently closed RNA molecules characterized by high stability and abundant expression across eukaryotes. They are increasingly recognized as key regulators in both physiological and pathological processes [4]. Canonical functions of circRNAs include acting as competitive endogenous RNAs (ceRNAs), protein scaffolds, and modifiers of mRNA splicing. Recent advances in genome-wide translational profiling, ribosome sequencing, mass spectrometry, and bioinformatics have unveiled the previously underappreciated protein-coding potential of circRNAs in cancer [5–7]. This has shifted research focus toward the roles of circRNA-encoded peptides or microproteins in tumorigenesis and therapy resistance, offering new avenues for biomarker discovery and targeted therapy. For instance, in hepatocellular carcinoma, circPETH1 encodes a 147-amino acid peptide via an m6A-dependent mechanism, driving metabolic reprogramming and shaping an immunosuppressive microenvironment [8]. Similarly, circZKSaa, a protein encoded by circZKSCAN1, sensitizes hepatocellular carcinoma cells to sorafenib by inhibiting the phosphatidylinositol 3-kinase (PI3K)/AKT/mechanistic target of rapamycin (mTOR) pathway [9]. In non-small cell lung cancer, the polypeptide C-IGF1R limits activated mitophagy and facilitates the transition of persister cancer cells toward apoptosis [10]. A limited number of studies have reported that circRNAs can serve as translational templates in GC, influencing various signaling pathways to regulate tumor cell proliferation, migration, invasion, and autophagy [11–14]. Nevertheless, whether circRNA-derived peptides or microproteins contribute to GC malignancy through other molecular mechanisms remains poorly understood.

Protein SUMOylation is a highly conserved and dynamic posttranslational modification involving the covalent attachment of small ubiquitin-like modifier (SUMO) proteins (SUMO1/2/3) to lysine residues on target substrates, thereby modulating their localization, stability, and function [15]. Growing evidence indicates that the SUMOylation network is aberrantly activated in many cancers [16]. Key enzymes such as SAE1 and UBC9 are overexpressed in multiple tumor types, enhancing the stability of oncogenic transcription factors like c-Myc and STAT3, thereby promoting proliferation and immune evasion. Moreover, SUMOylation regulates liquid–liquid phase separation of DNA repair proteins such as RNF168 and BRCA1, affecting nonhomologous end joining efficiency and contributing to genomic instability and chemoresistance [17,18].

Endoplasmic reticulum (ER) stress is a hallmark of the tumor microenvironment, and the unfolded protein response (UPR) serves as a central adaptive mechanism to mitigate proteostatic imbalance. The UPR is mediated through 3 major branches: IRE1α–XBP1, PERK–eIF2α, and ATF6, which collectively determine cell fate toward survival or apoptosis [19]. In GC, aberrant activation of the UPR is closely linked to chemotherapy resistance—XBP1s enhances 5-fluorouracil tolerance by up-regulating HSPA5 (GRP78), while ATF4 promotes glutathione synthesis via SLC7A11, suppressing ferroptosis [20]. However, current research has largely focused on classical UPR signaling components, with the regulatory roles of posttranslational modifications on key UPR factors remaining underexplored.

In this study, we integrated high-throughput data from transcriptomic and translatomic analyses to perform a multi-dimensional investigation, through which we identified a noncanonical open reading frame (ORF) embedded within circRNA circRAD23B and its encoded novel protein in GC. This protein, which we termed circRAD23B-208aa, was further investigated to elucidate its biological functions and molecular regulatory mechanisms in the development and progression of GC. Our findings uncover a previously unrecognized role of a circRNA-encoded protein in regulating proteostasis through posttranslational modification and highlight the circRAD23B-208aa/PDIA5/ATF6 axis as a promising therapeutic target for interfering with GC progression and overcoming treatment resistance.

## Results

### A coding-capable circRAD23B is implicated in GC progression

**Fig. 1.**
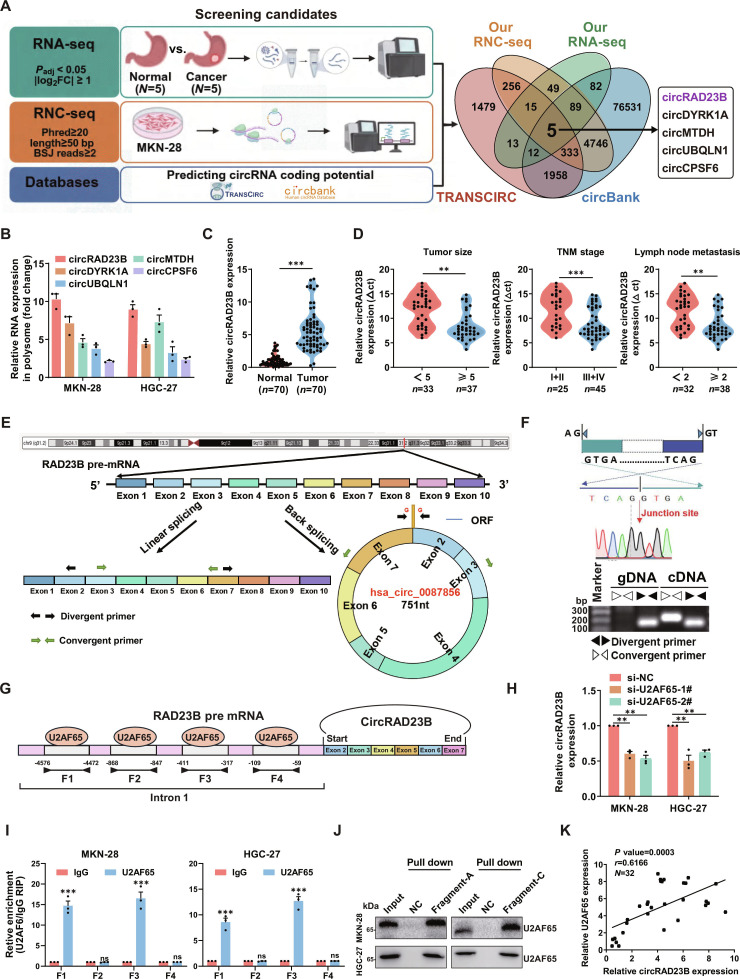
A coding-capable circRAD23B is implicated in GC progression. (A) Venn diagram analysis identifies circRNAs with coding potential from RNC-seq, RNA-seq, TransCirc, and Circbank databases (|log_2_(fold change)| ≥ 1, FDR < 0.05). (B) Ribosome fractionation assay demonstrates ribosome binding affinity of circRAD23B, circDYRK1A, circUBQLN1, circMTDH, and circCPSF6 in GC cells. (C) qRT-PCR detection of circRAD23B in 70 paired GC and adjacent normal tissues (paired samples, 2-sided Student’s *t* test). (D) Correlation between circRAD23B expression and clinicopathological features in 60 patients (2-sided Student’s *t* test). (E) Schematic of circRAD23B back-splicing formation. (F) Sanger sequencing validation of back-splice junction; agarose gel confirmation of circular product. (G) RBPsuite prediction of U2AF65 binding to RAD23B pre-mRNA intron 1. (H) qRT-PCR analysis of circRAD23B after U2AF65 knockdown (unpaired *t* test). (I) RIP assay confirms U2AF65 interaction with RAD23B pre-mRNA intron 1 (unpaired *t* test). (J) RNA pull-down validates U2AF65 binding to RAD23B pre-mRNA exon 1 regions A/C. (K) Correlation analysis between U2AF65 and circRAD23B in GC tissues (*n* = 32; Pearson correlation). Data presented as mean ± SD. **P < 0.01, ***P < 0.001.

To identify functional circRNAs with coding potential in GC, we integrated RNA sequencing (RNA-seq) data from 5 paired tumor and normal tissues with ribosome nascent-chain complex sequencing (RNC-seq) data from MKN-28 cells. Cross-referencing with TransCirc [21] and Circbank [22] databases revealed 5 up-regulated circRNAs predicted to encode proteins: circRAD23B, circDYRK1A, circMTDH, circUBQLN1, and circCPSF6 (Fig. 1A and Fig. S1A and B). Ribosomal fractionation in MKN-28 and HGC-27 cells indicated that circRAD23B had the highest ribosome binding, suggesting strong translation potential (Fig. 1B). Although circRAD23B was reported to promote bladder cancer progression via miR-1184 sponging, its role and coding capacity in GC remained unknown [23].

Building on the finding that circRAD23B was consistently up-regulated in multiple GC cell lines, we further confirmed its marked up-regulation in a cohort of 70 clinically matched GC tissue samples (Fig. 1C and Fig. S1C). This clinical relevance was reinforced by its important overexpression in 70 paired patient samples, where high circRAD23B levels correlated with larger tumor size, advanced tumor–node–metastasis (TNM) stage, and lymph node metastasis (Fig. 1D)

CircRAD23B (hsa_circ_0087856) arises from back-splicing of exons 2 to 7 of RAD23B (Fig. 1E). Its circular nature was confirmed by divergent primer-based polymerase chain reaction (PCR) from complementary DNA (cDNA) but not genomic DNA (gDNA), and Sanger sequencing verified the back-splice junction (Fig. 1F). circRAD23B also demonstrated greater stability than linear RAD23B under ribonuclease (RNase) R digestion and actinomycin D treatment (Fig. S1D and E).

We next investigated its biogenesis mechanism. Bioinformatics analysis via RBPsuite [24] predicted strong binding of splicing factor U2AF65 to 4 regions within intron 1 of RAD23B (Fig. 1G). Notably, high U2AF65 expression correlated with shorter progression-free survival (PFS), overall survival (OS), and post-progression survival (PPS) in GC patients based on publicly available databases (Fig. S2A). Knockdown of U2AF65 markedly impaired the proliferation, migration, and invasion of GC cells (Fig. S2B to E), indicating its oncogenic role. Consistently, depletion of U2AF65 markedly reduced circRAD23B expression (Fig. 1H). RNA immunoprecipitation (RIP) and RNA pull-down assays confirmed that U2AF65 directly binds fragments 1 and 3 of RAD23B pre-mRNA (Fig. 1I and J), indicating that U2AF65 facilitates circRAD23B cyclization. A strong positive correlation between U2AF65 and circRAD23B expression was further observed in 60 clinical samples (Fig. 1K). These findings collectively indicate that U2AF65 promotes GC malignancy at least in part by regulating circRAD23B biogenesis.

Functional assays using short hairpin RNAs (shRNAs) targeting the back-splice junction showed that knockdown of circRAD23B (sh-circRAD23B-2/3; Fig. S1F and G) markedly suppressed cell proliferation (Fig. S1H and I) and reduced migration and invasion (Fig. S1J), demonstrating its critical role in promoting malignant behaviors in GC.

### CircRAD23B encodes a novel protein circRAD23B-208aa to promote gastric tumorigenesis

**Fig. 2.**
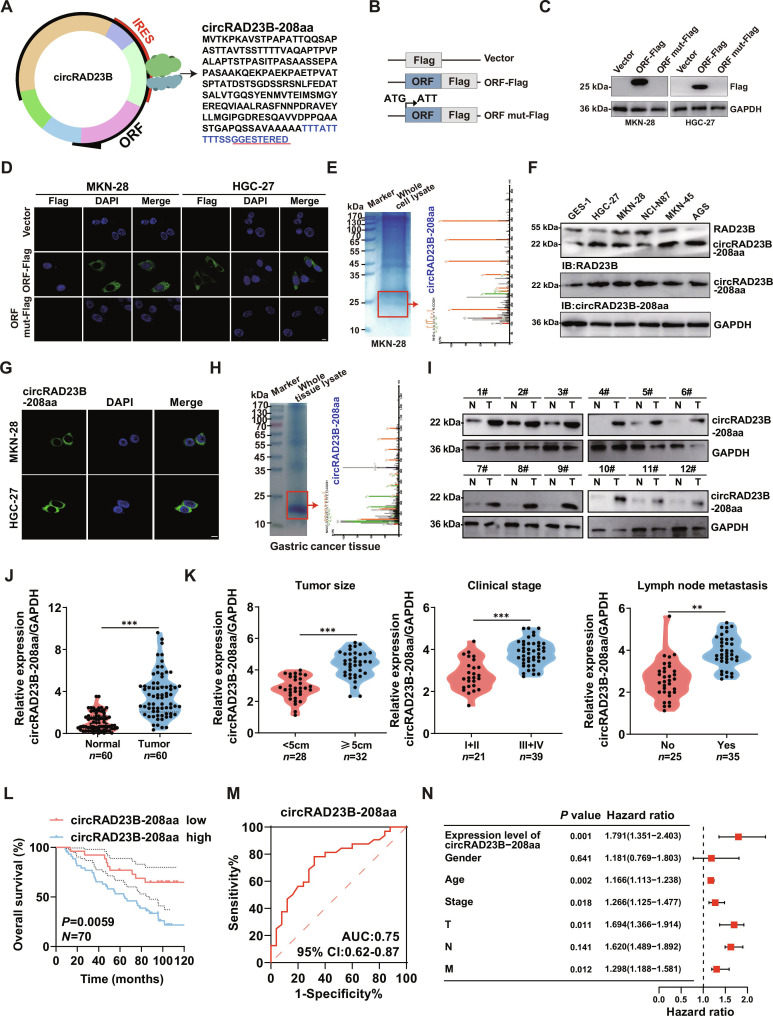
CircRAD23B encodes the novel protein circRAD23B-208aa to promote gastric tumorigenesis. (A) Schematic of circRAD23B-208aa ORF and IRES prediction. (B) Construct design of ORF with start codon mutants. (C) Western blot validation of ORF activity. (D) Immunofluorescence (IF) detects the activity of ORF and subcellular localization. Scale bars, 20 μm. (E) Coomassie blue staining of SDS-PAGE separated total proteins from MKN-28 cells (left); mass spectrometry identification of bands at target molecular weight (right). (F) Western blot analysis of circRAD23B-208aa and RAD23B expression in GC cells versus normal gastric mucosa. (G) IF detects the localization of circRAD23B-208aa in GC cells. Scale bars, 20 μm. (H) Mass spectrometry validation of endogenous circRAD23B-208aa in GC tissues. (I) Western blot analysis of circRAD23B-208aa and its parental RAD23B protein expression in 12 paired GC tumor (T) and adjacent normal (N) tissues. (J) Quantification of circRAD23B-208aa protein levels (normalized to GAPDH) in 60 paired GC and adjacent normal tissues. Data were analyzed using a paired 2-tailed Student’s *t* test. (K) Correlation between circRAD23B-208aa protein expression and clinicopathological features, including tumor size, clinical stage, and lymph node metastasis, in 60 GC patients. Data were analyzed using a 2-tailed Student’s *t* test. (L) Kaplan–Meier survival analysis of OS in 60 GC patients stratified by high or low circRAD23B-208aa protein expression. The log-rank test was used to calculate statistical significance. (M) ROC curve analysis of circRAD23B-208aa expression for distinguishing GC tissues from adjacent normal tissues. The AUC value is indicated. (N) Multivariate Cox regression analysis of OS in 60 GC patients. The forest plot displays the hazard ratio (HR) and 95% confidence interval (CI) for circRAD23B-208aa expression and other clinicopathological parameters. Data presented as mean ± SD. **P < 0.01, ***P < 0.001.

To investigate the coding potential of circRAD23B, we analyzed its sequence and identified an ORF of 627 nucleotides containing an internal ribosome entry site (IRES), suggesting its potential to encode a protein of 208 amino acids (Fig. 2A). To test this hypothesis, we constructed a plasmid harboring this ORF tagged with a Flag epitope, along with a control plasmid carrying a mutation in the start codon (ATG). Western blot and immunofluorescence assays detected the ORF-Flag fusion protein, while the signal was abolished upon ATG mutation, providing initial evidence for the translational activity of this start codon (Fig. 2B to D).

To further confirm that circRAD23B-208aa is endogenously expressed in GC rather than being a cleavage product of the parental RAD23B protein, we generated a custom antibody targeting a unique epitope within the circRAD23B ORF that is absent in RAD23B (underlined in Fig. 2A). This antibody detected a single band of approximately 22 kDa in GC cell lines, consistent with the predicted size (Fig. 2F and Fig. S2F). Endogenous expression of circRAD23B-208aa was also observed via immunofluorescence (Fig. 2G). We further excised gel segments corresponding to ~22 kDa from Coomassie blue-stained sodium dodecyl sulfate–polyacrylamide gel electrophoresis (SDS-PAGE) gels of MKN-28 cells and GC tissues for mass spectrometry analysis, which successfully identified specific peptides unique to circRAD23B-208aa (Fig. 2E and H).

Western blot analysis of 60 paired GC and adjacent normal tissues revealed important up-regulation of circRAD23B-208aa in tumor tissues (Fig. 2I and J and Fig. S2J). Although densitometric comparison does not establish absolute abundance, these data indicate that circRAD23B-208aa is detectable in GC cells at levels sufficient to support functional effects in our assays. Clinicopathological correlation analysis indicated that high circRAD23B-208aa expression was associated with larger tumor size, advanced TNM stage, lymph node metastasis, and shorter OS in GC patients (Fig. 2K and L).

#### circRAD23B-208aa expression distinguishes tumor from adjacent normal tissue in the same cohort

As a descriptive summary, receiver operating characteristic (ROC) curve analysis gave an area under the curve (AUC) of 0.75 for distinguishing tumor tissues from adjacent normal tissues in our cohort (Fig. 2M). This finding simply indicates that circRAD23B-208aa expression levels differ between the 2 tissue types; no claim of diagnostic utility is made.

#### Exploratory survival association

In univariate analysis, circRAD23B-208aa expression showed a potential association with OS (Fig. 2N). In this small cohort (*n* = 60, with 22 death events), the number of events per variable was limited. Proportional hazards assumption was tested and met. Given these constraints and the exploratory nature of the analysis, these results should be considered preliminary and require validation in larger independent cohorts. We acknowledge that these findings are not sufficient to claim independent prognostic value.

Together, these findings indicate that circRAD23B-208aa is up-regulated in GC and correlates with malignant progression, warranting further validation in larger, independent cohorts.

Chen and Sarnow [25] previously demonstrated using in vitro translation systems that circRNAs containing IRES elements can be efficiently translated by eukaryotic ribosomes, whereas those lacking IRES fail to produce detectable protein products. We therefore hypothesized that the IRES within circRAD23B mediates translation of its embedded ORF. Dual-luciferase reporter assays showed that mutation or truncation of the IRES markedly suppressed circRAD23B-208aa translation in a dose-dependent manner, confirming IRES-dependent translation (Fig. S2G to I).

**Fig. 3.**
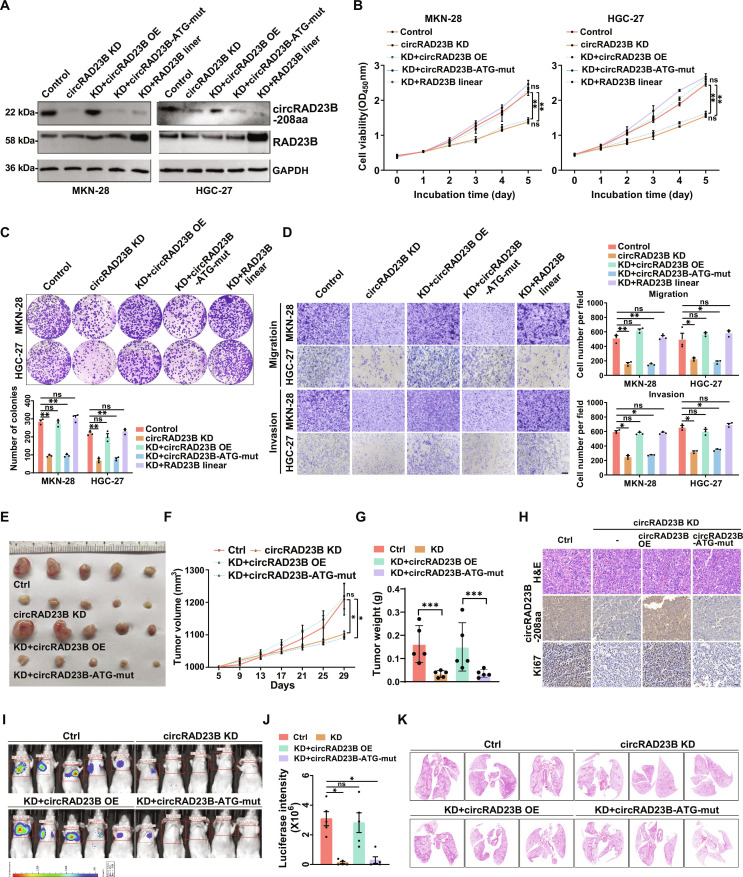
circRAD23B-208aa promotes GC cell proliferation and metastasis in vitro and in vivo. (A) Western blot analysis of circRAD23B knockdown and mutant rescue efficiency. (B) Cell proliferation by CCK-8 assay (*n* = 5; 2-way ANOVA). (C) Colony formation assay evaluating proliferative capacity (*n* = 3; unpaired *t* test). (D) Transwell assays detecting migration and invasion (*n* = 5; unpaired *t* test). Scale bars, 50 μm. (E) Representative images of xenograft tumors from the CDX mouse model in each indicated group. Scale bar, 1 cm. (F) Tumor growth curves measured at the indicated time points after subcutaneous injection (*n* = 5 per group). Data are presented as mean ± SD. Statistical analysis was performed using 2-way ANOVA. (G) Tumor weights of excised xenografts from each group at the experimental endpoint. Data are presented as mean ± SD (*n* = 5). Statistical analysis was performed using unpaired 2-tailed Student’s *t* test. (H) Immunohistochemical (IHC) staining of circRAD23B-208aa and Ki67 in xenograft tumor tissues from the CDX mouse model in each indicated group. Representative images are shown. Scale bar, 50 μm. (I and J) Tail-vein experimental lung colonization assay. Representative bioluminescence images of metastatic tumor burden (I) and quantification of fluorescence signals (J) for each indicated group (*n* = 5 per group). (K) Representative H&E staining of lung sections showing metastatic nodules. Scale bars, 200 μm. Data presented as mean ± SD. *P < 0.05; **P < 0.01; ns, not significance.

To investigate the biological function of circRAD23B-208aa, we constructed vectors containing flanking circularization sequences to generate the following stable cell lines: empty vector control, circRAD23B for expressing circularized RNA, circRAD23B-ATG-mut (start codon mutated to ATT) that forms circRNA but fails to translate the protein, and circRAD23B-linear lacking the circularization elements. Overexpression of circRAD23B-208aa in MKN-28 and HGC-27 cells was confirmed by Western blot (Fig. 3A). Cell Counting Kit-8 (CCK-8), and colony formation assays demonstrated that circRAD23B-208aa, but not the circRAD23B RNA itself, markedly promoted GC cell proliferation (Fig. 3B and C). Transwell assays further revealed that circRAD23B-208aa enhanced cell migration and invasion (Fig. 3D). These results indicate that circRAD23B facilitates GC cell proliferation and invasion in vitro through its encoded protein product. To exclude the possibility that the IRES element itself might influence cell function independently of its role in translation, we performed additional control experiments. MKN-28 cells ectopically expressing IRES-WT, IRES-Mut, or IRES-Δ constructs were subjected to functional assays. As shown in Fig. S3A to C, neither mutation nor truncation of the IRES sequence affected GC cell proliferation, migration, or invasion, indicating that the IRES element alone does not contribute to the malignant phenotypes observed.

We next evaluated the in vivo oncogenic role of circRAD23B-208aa using xenograft tumor models. MKN-28 cells stably expressing the indicated constructs were subcutaneously injected into nude mice. Tumor volume measurements over time and tumor weights at the experimental endpoint showed that circRAD23B-208aa overexpression markedly promoted tumor growth, whereas the translation-deficient mutant failed to exert this effect (Fig. 3E to G). Immunohistochemical staining of xenograft tissues confirmed elevated expression of circRAD23B-208aa and the proliferation marker Ki67 in the circRAD23B-208aa overexpression group (Fig. 3H). Furthermore, in a tail-vein injection model, circRAD23B-208aa markedly enhanced the pulmonary seeding and colonization capacity of HGC-27 cells in lung tissue, as evidenced by bioluminescence imaging and hematoxylin and eosin (H&E) staining of lung sections (Fig. 3I to K). It should be noted that the tail-vein injection model bypasses the initial steps of local invasion and intravasation, and therefore reflects experimental lung colonization rather than spontaneous metastatic progression. In this exploratory setting (*n* = 5 per group), we observed a important reduction in lung colonization in the sh-circ group, suggesting a potential role of circRAD23B-208aa in promoting experimental lung colonization. These findings warrant validation in larger, pre-powered studies. Collectively, these findings demonstrate that circRAD23B-208aa promotes GC proliferation and invasion.

### CircRAD23B-208aa stabilizes PDIA5 protein by facilitating its SUMOylation

**Fig. 4.**
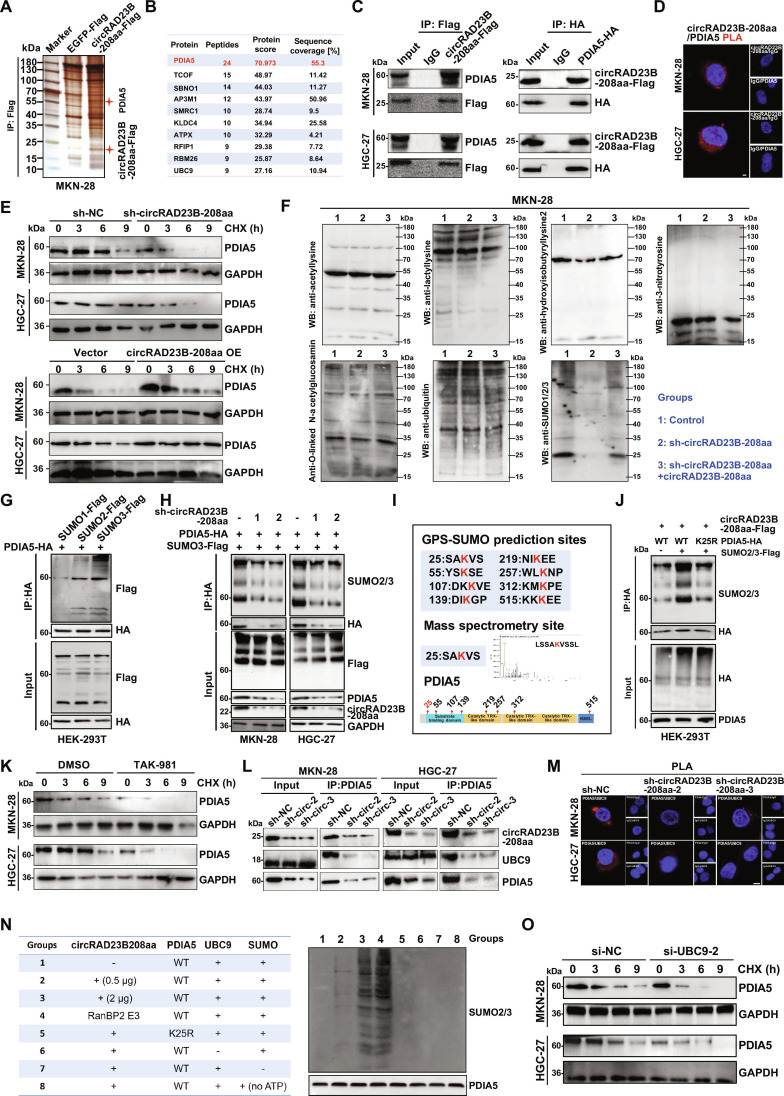
CircRAD23B-208aa stabilizes PDIA5 protein by facilitating its SUMOylation​​. (A) Silver staining of proteins interacting with circRAD23B-208aa-Flag. (B) List of high-scoring PDIA5-bound proteins identified by mass spectrometry. (C and D) Co-IP (C) and PLA (D) validating PDIA5–circRAD23B-208aa interaction. Scale bars, 20 μm. (E) PDIA5 half-life analysis post-circRAD23B-208aa modulation. (F) Knock down circRAD23B-208aa in MKN-28 cells, or knock down circRAD23B-208aa followed by its re-overexpression, and then measure the alterations in protein posttranslational modification levels. (G) Co-IP detection of PDIA5 and SUMO1/SUMO2/SUMO3 abundance. (H) Reduced SUMO2/3 binding to PDIA5 post-circRAD23B-208aa knockdown. (I) GPS-SUMO prediction of PDIA5 SUMOylation sites (top); mass spectrometry-identified sites (bottom). (J) Mutation of PDIA5 K25 residue abolishes SUMOylation. (K) Decreased PDIA5 half-life following TAK-981 treatment. (L and M) Co-IP (L) and PLA (M) validating reduced PDIA5-UBC9 binding after circRAD23B-208aa knockdown. (N) In vitro SUMOylation assay showing that circRAD23B-208aa promotes SUMOylation of PDIA5 in a dose- and UBC9-dependent manner. (O) Decreased PDIA5 half-life upon UBC9 knockdown.

To elucidate the mechanism by which circRAD23B-208aa promotes GC progression, we first performed RNA-seq on MKN-28 cells with stable knockdown of circRAD23B-208aa. The results revealed that differentially expressed genes were primarily enriched in pathways such as the UPR (Fig. S6A and B). Concurrently, we conducted co-immunoprecipitation (Co-IP) followed by mass spectrometry in MKN-28 cells stably overexpressing circRAD23B-208aa-Flag to identify its interacting proteins in GC (Fig. 4A). Based on protein score, sequence coverage, and literature review, PDIA5 (protein disulfide isomerase family A member 5) emerged as a candidate of interest (Fig. 4B and Fig. S4A to C). Their interaction was further validated by Co-IP and proximity ligation assays (PLAs) in both cultured cells and xenograft tumor tissues (Fig. 4C and D and Fig. S4D). Truncation mapping experiments indicated that the N-terminal region of circRAD23B-208aa interacts with domain 1 of PDIA5 (Fig. S4E and F).

PDIA5, a member of the protein disulfide isomerase (PDI) family, has been identified as a key regulator of the ER stress response in glioma [26]. It promotes tumor cell survival and chemoresistance under stress conditions by modulating ATF6 activation and downstream signaling, suggesting that targeting the PDIA5/ATF6 axis may offer a strategy to overcome therapy resistance [27]. Ji et al. [28] reported that in glioblastoma cells, PDIA5 is transcriptionally up-regulated by RUNX1, leading to increased PDIA5 protein expression, which in turn facilitates cell proliferation and invasion by promoting the proper folding and maturation of CCAR1. Cheng and colleagues [26,29] documented aberrant PDIA5 expression in several human cancers, highlighting its dual role as a regulator of proteostasis and a potential biomarker. Additionally, PDIA5 regulates fluorouracil chemoresistance in colorectal cancer cells by inhibiting RNASET2-mediated RNA degradation and modulating uracil metabolism [30]. However, the function and mechanism of PDIA5 in GC have remained unexplored.

To investigate how circRAD23B-208aa influences GC progression via PDIA5, we knocked down circRAD23B-208aa and observed a subsequent decrease in PDIA5 protein levels by Western blot, while its mRNA levels and subcellular localization remained unchanged (Fig. S4G to I). Protein stability assays showed a shortened half-life of PDIA5 upon circRAD23B-208aa depletion, suggesting posttranslational regulation of PDIA5 stability by circRAD23B-208aa (Fig. 4E). We then screened several common posttranslational modifications using pan antibodies against lactylation, acetylation, nitrosylation, SUMOylation, ubiquitination, and glycosylation in MKN-28 cells with stable knockdown of circRAD23B-208aa and subsequent rescue expression. Results indicated that only SUMOylation levels changed markedly with circRAD23B-208aa expression (Fig. 4F). Prediction using GPS-SUMO 2.0 [31] suggested 10 potential lysine sites for SUMOylation on PDIA5 (Fig. 4I), implying that circRAD23B-208aa might regulate PDIA5 stability via SUMOylation. SUMO-IP assays confirmed that PDIA5 is modified by SUMO, primarily by SUMO3 (Fig. 4G), and this modification was attenuated upon circRAD23B-208aa knockdown (Fig. 4H). Further mass spectrometric analysis of SUMOylation identified a potential modification site at K25 of PDIA5 (Fig. 4I). Mutation of lysine 25 to arginine (K25R) markedly reduced SUMOylation levels (Fig. 4J). Treatment with the SUMOylation inhibitor TAK-981 shortened the half-life of PDIA5 (Fig. 4K). These results demonstrate that circRAD23B-208aa enhances SUMOylation of PDIA5 at K25, thereby maintaining its protein stability.

UBC9 is the only identified E2 conjugating enzyme for SUMOylation [32]. We therefore investigated whether UBC9 mediates PDIA5 SUMOylation. Reanalysis of the circRAD23B-208aa immunoprecipitation–mass spectrometry (IP-MS) data identified UBC9 as a candidate interacting partner. Two-step Co-IP and multiplex immunofluorescence confirmed pairwise interactions among circRAD23B-208aa, PDIA5, and UBC9 (Fig. S4J and K). Moreover, knockdown of circRAD23B-208aa weakened the interaction between PDIA5 and UBC9 (Fig. 4L and M). To verify whether circRAD23B-208aa enhances the formation of the circRAD23B-208aa/UBC9/PDIA5 complex and the SUMOylation modification of PDIA5, we performed in vitro SUMOylation assays using purified recombinant proteins. Addition of circRAD23B-208aa enhanced PDIA5 SUMOylation in a dose-dependent manner, whereas the PDIA5-K25R mutant abolished this enhancement (Fig. 4N). Furthermore, AlphaFold Server-based protein docking predicted that circRAD23B-208aa (blue) contacts both PDIA5 (pink) and UBC9 (green) through hydrogen bonds (yellow dashed lines; Fig. S4L). This computational prediction is hypothetical and requires experimental validation. To validate this regulatory axis in vivo, we examined PDIA5 SUMOylation levels in subcutaneous xenograft tumors following circRAD23B-208aa knockdown. Co-IP assays using an anti-PDIA5 antibody followed by anti-SUMO2/3 Western blotting revealed markedly reduced PDIA5 SUMOylation in sh-circ tumors compared with sh-NC controls (Fig. S4M). Depletion of UBC9 reduced PDIA5 SUMOylation, shortened its half-life, and decreased its stability (Fig. 4O and Fig. S4N). These findings indicate that circRAD23B-208aa promotes PDIA5 protein stability through UBC9-mediated SUMOylation.

Furthermore, analysis of 58 paired GC tissues revealed marked up-regulation of PDIA5 in tumors, and its expression level was positively correlated with that of circRAD23B-208aa (Fig. S4O to Q). In summary, circRAD23B-208aa facilitates PDIA5 SUMOylation at K25 and stabilizes PDIA5, which is consistent with a scaffold-like function but does not prove it directly.

### CircRAD23B-208aa antagonizes STUB1-mediated PDIA5 ubiquitination and subsequent degradation

**Fig. 5.**
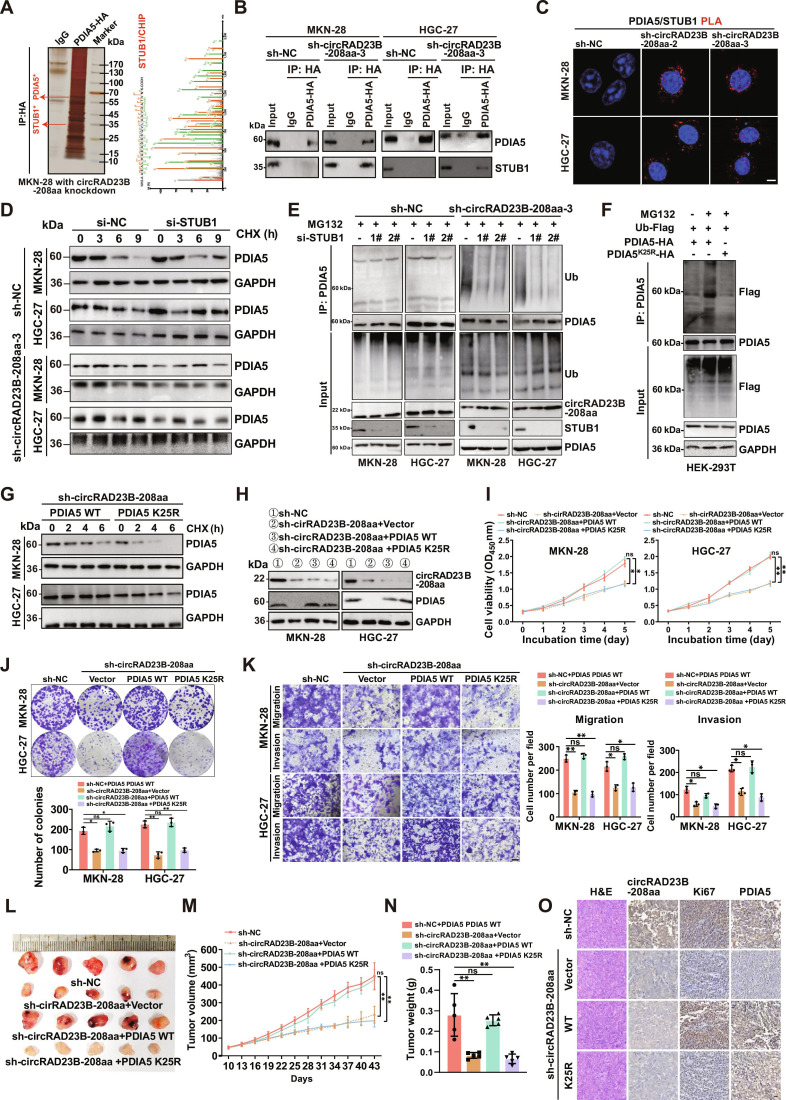
CircRAD23B-208aa antagonizes STUB1-mediated PDIA5 ubiquitination and subsequent degradation.​ (A) Silver staining of PDIA5-HA interacting proteins after circRAD23B-208aa knockdown. (B and C) Co-IP (B) and PLA (C) showing increased PDIA5-STUB1 binding post-circRAD23B-208aa knockdown. Scale bars, 20 μm. (D) Decreased PDIA5 half-life following STUB1 knockdown. (E) Decreased PDIA5 ubiquitination after STUB1 knockdown. (F) Ubiquitination assays of wild-type (WT) PDIA5 and K25R mutant PDIA5 in HEK-293T cells cotransfected with HA-ubiquitin and STUB1. PDIA5 was immunoprecipitated with anti-PDIA5 antibody, and ubiquitination levels were detected with anti-Flag antibody. (G) Cycloheximide chase analysis of PDIA5 half-life in circRAD23B-208aa-knockdown GC cells transduced with empty vector, wild-type PDIA5, or the PDIA5 K25R mutant. (H) Western blot validation of rescue efficiency in the indicated groups. (I) Cell proliferation was assessed by CCK-8 assays at the indicated time points. Data are presented as mean ± SD (*n* = 5). Statistical analysis was performed using 2-way ANOVA. (J) Colony formation assays evaluating proliferative capacity. Representative images (left) and quantification (right) are shown. Data are presented as mean ± SD (*n* = 3). (K) Transwell migration and invasion assays. Representative images (left) and quantification of migrated or invaded cells per field (right) are shown. Scale bar, 100 μm. Data are presented as mean ± SD (*n* = 5), unpaired 2-tailed Student’s *t* test. (L to N) In vivo validation of the PDIA5 K25R mutant in rescue experiments. Representative images of xenograft tumors from the CDX mouse model in each indicated group (L). Tumor growth curves measured at the indicated time points (M) and tumor weights at the experimental endpoint (N) are shown. Data are presented as mean ± SD (*n* = 5 per group). Statistical analysis was performed using 2-way ANOVA for (M) and unpaired 2-tailed Student’s *t* test for (N). (O) IHC staining of circRAD23B-208aa, Ki67, and PDIA5 in xenograft tumor tissues from the indicated rescue groups of the CDX mouse model. Representative images are shown. Scale bar, 50 μm. Data presented as mean ± SD. *P < 0.05; **P < 0.01; ns, no significance.

In eukaryotic cells, protein stability is primarily regulated by the ubiquitin–proteasome system, in which E3 ubiquitin ligase-mediated ubiquitination serves as a central signal for targeted protein degradation [33]. Accumulating evidence indicates extensive crosstalk between SUMOylation and the ubiquitination pathway, wherein SUMOylation often stabilizes target proteins by antagonizing their ubiquitination [34]. To investigate whether circRAD23B-208aa maintains PDIA5 stability by promoting its SUMOylation and thereby counteracting ubiquitin-dependent degradation, we first performed Co-IP of PDIA5 followed by mass spectrometry in MKN-28 cells with stable knockdown of circRAD23B-208aa to identify PDIA5-interacting proteins. Analysis of the proteomic data revealed the E3 ubiquitin ligase STUB1/CHIP (Fig. 5A). Subsequent Co-IP and PLA demonstrated that PDIA5 barely interacted with STUB1/CHIP when circRAD23B-208aa was abundant, whereas their binding increased upon circRAD23B-208aa knockdown (Fig. 5B and C). Moreover, inhibition of STUB1/CHIP expression in the context of circRAD23B-208aa depletion restored PDIA5 stability and reduced its ubiquitination levels (Fig. 5D and E and Fig. S5A). Importantly, introducing a K25R mutation at the SUMOylation site of PDIA5 under circRAD23B-208aa knockdown conditions almost completely abolished PDIA5 ubiquitination (Fig. 5F). These findings suggest that circRAD23B-208aa-mediated SUMOylation of PDIA5 stabilizes the protein by antagonizing its ubiquitination.

We further explored the biological function of PDIA5. CCK-8 and colony formation assays showed that knockdown of PDIA5 markedly impaired the proliferation of GC cells (Fig. S5B and C). Transwell assays indicated that suppression of PDIA5 reduced migratory and invasive capacities (Fig. S5D). PROTEOSTAT and thioflavin T (ThT) staining revealed an accumulation of misfolded proteins and suppression of the UPR upon PDIA5 knockdown (Fig. S5E and F). Consistently, Western blot analysis showed that PDIA5 knockdown increased the expression of the endoplasmic reticulum stress markers binding immunoglobulin protein/glucose-regulated protein 78 (BiP/GRP78) and C/EBP homologous protein (CHOP) in gastric cancer cells. PDIA5 knockdown also reduced intracellular adenosine triphosphate (ATP) levels. (Fig. S5G and H). Scanning electron microscopy further showed marked swelling and dilation of the ER in PDIA5-deficient cells, indicating enhanced ER stress (Fig. S5I). Similar effects were observed upon knockdown of circRAD23B-208aa (Fig. S6E to J).

Therefore, PDIA5 plays an essential role in maintaining ER homeostasis, which is requisite for the aggressive phenotypes of GC cells. We next performed rescue experiments to determine whether circRAD23B-208aa promotes GC malignancy through PDIA5 SUMOylation. PDIA5 wild-type or K25R mutant was reexpressed in MKN-28 cells stably expressing sh-circRAD23B-208aa. Protein stability assays revealed that wild-type PDIA5 exhibited a longer half-life compared to the K25R mutant upon circRAD23B-208aa knockdown (Fig. 5G). Functionally, reexpression of wild-type PDIA5 markedly rescued the proliferation, migration, and invasion defects caused by circRAD23B-208aa knockdown, whereas the K25R mutant failed to do so (Fig. 5H to K). In vivo, xenograft tumor assays confirmed that only wild-type PDIA5 restored tumor growth in circRAD23B-208aa-depleted cells (Fig. 5L to N). Immunohistochemical staining further showed that only wild-type PDIA5 rescued the expression of the proliferation marker Ki67 (Fig. 5O). Collectively, these results demonstrate that circRAD23B-208aa stabilizes PDIA5 through UBC9-mediated SUMOylation, thereby protecting it from STUB1-dependent ubiquitination and degradation to promote GC progression.

### PDIA5 promotes ATF6 cleavage and nuclear translocation

**Fig. 6.**
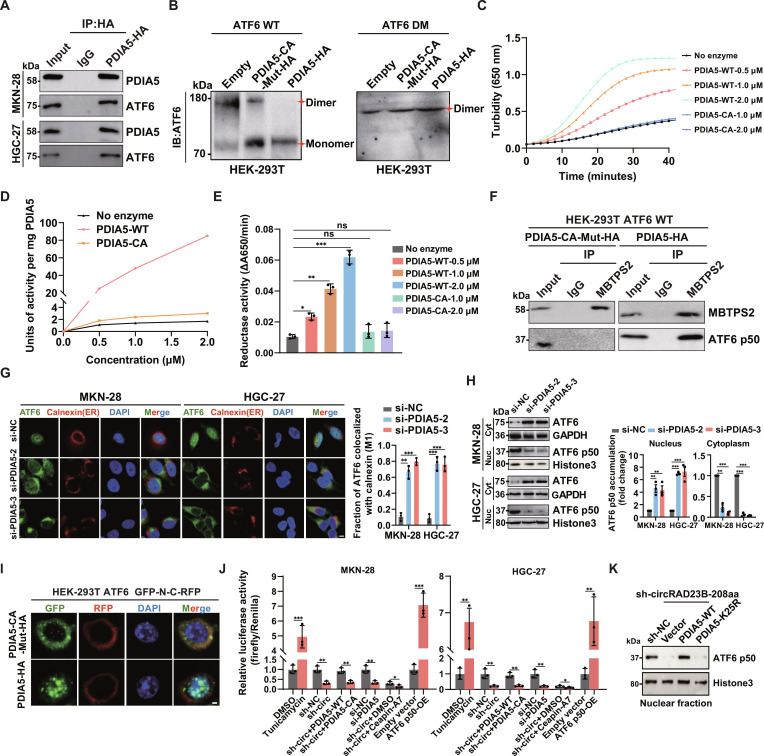
PDIA5 promotes ATF6 cleavage and nuclear translocation. (A) Co-IP analysis showing the direct interaction between PDIA5 and ATF6 in GC cells. (B) After transfection of PDIA5-HA and its CA mutants in HEK-293T cells, the level of ATF6 was detected in a nonreducing state. (C) Time course of insulin-induced turbidity (A650) catalyzed by PDIA5-WT or PDIA5-CA at indicated concentrations. (D) Quantification of reductase activity (ΔA650/min) derived from the linear phase. (E) Specific activity (units per mg PDIA5) of PDIA5-WT and PDIA5-CA compared to no-enzyme control. (F) Co-IP shows the binding of MBTPS2 to ATF6 p50 after transfection with PDIA5 or its CA mutant. (G) IF shows the degree of overlap between ATF6 and ER after PDIA5 knocking down. Scale bar, 20 μm. (H) Nuclear-cytoplasmic fractionation showing ATF6 nuclear accumulation post-PDIA5 knockdown. (I) IF images demonstrating the nuclear localization of the cleaved ATF6 p50 fragment upon coexpression with wild-type PDIA5, but not the PDIA5-CA mutant. (J) ERSE luciferase reporter assay measuring ATF6-driven transcription. (K) Nuclear fractionation Western blot detecting ATF6 p50 levels in nuclear extracts. Western blot of nuclear ATF6 p50 in subcutaneous xenograft tumors (from the model in Fig. 5L). Histone 3 serves as a nuclear loading control. Data are presented as mean ± SD (*n* = 3). Data presented as mean ± SD. *P < 0.05; **P < 0.01; ***P < 0.001; ns, not significance.

Given the established role of PDIA5 in regulating the UPR and its potential involvement in GC malignant progression, Higa et al. [27] proposed that PDIA5 facilitates ATF6 nuclear translocation by catalyzing disulfide bond rearrangement. Based on this evidence, we hypothesized that elevated PDIA5 expression may contribute to ATF6α activation—specifically by promoting its reductive activation, facilitating dissociation from the ER, and enabling subsequent Golgi processing and nuclear translocation, ultimately leading to transcriptional activation. To test this, we first confirmed the physical interaction between PDIA5 and ATF6 by Co-IP (Fig. 6A). Overexpression of wild-type PDIA5, but not its catalytically inactive CA mutant (C53S/C56S), promoted the reduction of ATF6 dimers to monomers in HEK-293T cells, an effect abolished by an ATF6 substrate-binding mutant (ATF6 DM), demonstrating that PDIA5 could influence ATF6 activation (Fig. 6B). Conversely, knockdown of circRAD23B-208aa prevented complete ATF6 monomerization (Fig. S6C). To rule out the possibility that this axis might also influence the other 2 arms of the UPR, we quantified the expression of key downstream target genes of the IRE1–XBP1 and PERK–eIF2α pathways by quantitative reverse transcription PCR (qRT-PCR). As shown in Fig. S6D, knockdown of circRAD23B-208aa did not markedly alter the mRNA levels of XBP1s, EDEM1, Sec61a1 (IRE1 axis) or ATF4, CHOP, ASNS, CHAC1 (PERK axis), whereas tunicamycin treatment, as a positive control, strongly induced all these targets. These results demonstrate that the circRAD23B-208aa/PDIA5 axis can regulate the ATF6 activation without engaging IRE1 or PERK signaling.

**Fig. 7.**
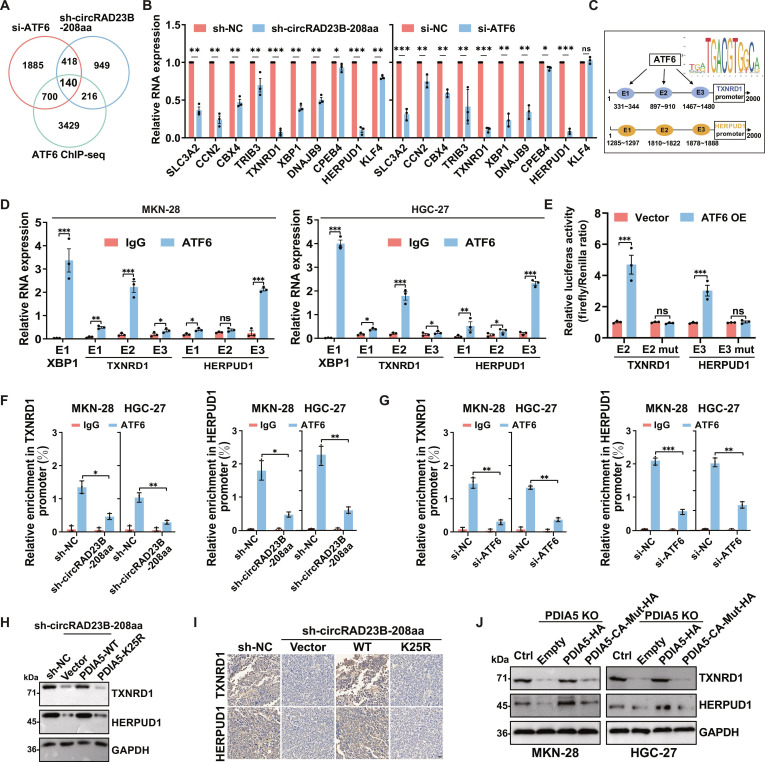
ATF6 activates TXNRD1 and HERPUD1 transcription to sustain UPR signaling. (A) Venn diagram of co-up-regulated genes after ATF6 or circRAD23B-208aa knockdown overlapping ATF6 ChIP-seq targets. (B) qRT-PCR validation of TXNRD1 and HERPUD1 expression changes. (C) ATF6 binding sites in TXNRD1(E2) and HERPUD1(E3) promoters. (D) ChIP-qPCR confirming ATF6 binding to promoters (XBP1E1 as positive control). (E) Dual-luciferase assay showing reduced promoter activity upon E2/E3 mutation despite ATF6 overexpression. (F and G) ChIP-qPCR assays showing that circRAD23B-208aa promotes ATF6 binding to the TXNRD1 (F) and HERPUD1 (G) promoters. (H) Western blot of TXNRD1 and HERPUD1 in subcutaneous xenograft tumors (Fig. 5L). (I) IHC staining of TXNRD1 and HERPUD1 in xenograft tumor tissues from the CDX mouse model in Fig. 5L. Representative images are shown. Scale bar, 50 μm. (J) Western blot analysis of TXNRD1 and HERPUD1 expression in the indicated rescue groups. Data presented as mean ± SD. *P < 0.05; **P < 0.01; ***P < 0.001; ns, no significance.

Furthermore, the results of insulin turbidity assay showed that recombinant PDIA5-WT exhibited strong, concentration-dependent activity in reducing insulin disulfide bonds, whereas the catalytically inactive PDIA5-CA mutant lost this ability. Upon reduction and dissociation from the ER, ATF6 is processed in the Golgi by S1P and S2P proteases [35]. In the presence of wild-type PDIA5, but not its catalytically inactive CA mutant, ATF6 interacted with the S2P protease MBTPS2 and was cleaved to release the p50 fragment for nuclear translocation (Fig. 6F). Conversely, PDIA5 knockdown markedly increased ATF6 retention in the ER (Fig. 6G). Accordingly, nuclear-cytoplasmic fractionation assays demonstrated that PDIA5 depletion markedly reduced nuclear localization of the ATF6 p50 fragment (Fig. 6H and I). ERSE luciferase assays (Fig. 6J) revealed that circRAD23B-208aa or PDIA5 knockdown reduced ATF6 transcriptional activity, which was rescued by PDIA5-WT but not by CA or K25R mutants. Consistently, in subcutaneous tumors, nuclear ATF6 p50 levels were decreased upon circRAD23B-208aa knockdown and restored by PDIA5-WT, but not by PDIA5-K25R (Fig. 6K). Functionally, reexpression of wild-type PDIA5, but not the CA mutant, rescued the proliferation, migration, and invasion defects caused by PDIA5 knockout in GC cells (Fig. 7A to D). Together, these findings support a model wherein circRAD23B-208aa enhances PDIA5 SUMOylation and protein stability, which in turn promotes ATF6 reduction, Golgi processing, and nuclear translocation specifically through the ATF6 branch of the UPR.

### ATF6 activates TXNRD1 and HERPUD1 transcription to sustain UPR signaling

To identify downstream transcriptional targets of nuclear ATF6, we performed transcriptome sequencing in GC cells with stable knockdown of either circRAD23B-208aa or ATF6. By integrating these data with a public ATF6 chromatin immunoprecipitation sequencing (ChIP-seq) dataset, we identified 140 overlapping candidate target genes (Fig. 7A). From these, we selected 10 differentially expressed genes implicated in the UPR for further validation. Among them, TXNRD1 and HERPUD1 exhibited the most pronounced reduction in expression upon circRAD23B-208aa or ATF6 knockdown (Fig. 7B). TXNRD1 (thioredoxin reductase 1) facilitates correct disulfide bond formation in the ER through the NADPH (reduced form of nicotinamide adenine dinucleotide phosphate)-dependent thioredoxin system, thereby promoting proper folding of secretory proteins. Its inhibition leads to accumulation of aberrant disulfide bonds, impaired secretion, and activation of ER stress responses [36]. HERPUD1 is an ER membrane protein and a core component of the HRD1 complex. It recruits DERL2 to facilitate retrotranslocation and degradation of misfolded proteins, playing a critical role in the HRD1–SEL1L-mediated ER-associated degradation pathway for both glycosylated and nonglycosylated substrates [37]. Both genes act as UPR-responsive effectors that alleviate ER stress through distinct mechanisms, suggesting that ATF6 may promote GC cell survival by activating TXNRD1 and HERPUD1 transcription.

To validate direct transcriptional regulation by ATF6, we analyzed the promoter regions of TXNRD1 and HERPUD1 using JASPAR [38], which predicted 3 potential ATF6 binding sites in each promoter (Fig. 7C). Subsequent ChIP-PCR assays confirmed that ATF6 predominantly binds to segment E2 of the TXNRD1 promoter and segment E3 of the HERPUD1 promoter (Fig. 7D). Mutating these binding sites abolished ATF6-mediated transactivation in dual-luciferase reporter assays (Fig. 7E), confirming their functional importance. Furthermore, ChIP-qPCR assays revealed that knockdown of either circRAD23B-208aa or ATF6 markedly reduced ATF6 occupancy at the TXNRD1 and HERPUD1 promoters (Fig. 7F and G). Consistently, reexpression of wild-type PDIA5, but not the SUMOylation-deficient K25R mutant or the catalytically inactive CA mutant, rescued TXNRD1 and HERPUD1 protein levels in circRAD23B-208aa or PDIA5 knockdown cells (Fig. 7H to J). These results indicate that circRAD23B-208aa-mediated PDIA5 SUMOylation and its enzymatic activity are both required for optimal ATF6 transcriptional activation of its downstream targets.

We next investigated the functional roles of TXNRD1 and HERPUD1 in GC cells. Knockdown of either gene markedly impaired cell proliferation, as shown by CCK-8 and colony formation assays (Fig. S7E to G and I to K). Transwell migration and invasion assays further revealed reduced metastatic potential upon TXNRD1 or HERPUD1 suppression (Fig. S7H and L). PROTEOSTAT and ThT staining indicated accumulation of misfolded proteins and dampened UPR signaling following knockdown (Fig. S8A and B). Consistently, Western blot analysis showed that knockdown of TXNRD1 or HERPUD1 up-regulated the expression of ER stress markers BiP/GRP78 and CHOP, and reduced intracellular ATP levels in GC cells (Fig. S8C and D).

**Fig. 8.**
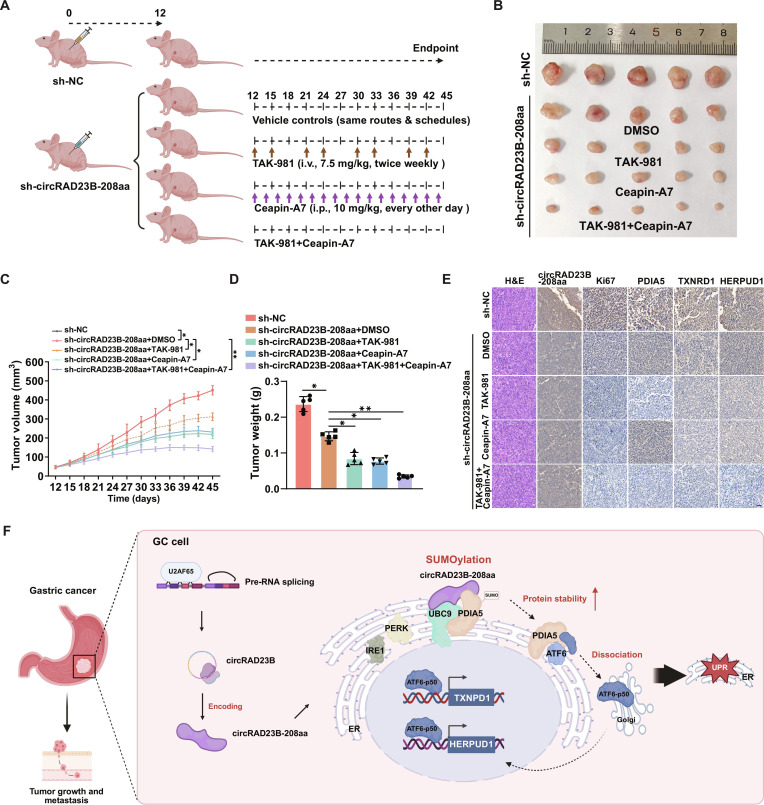
Pharmacological inhibition of SUMOylation and ATF6 markedly suppresses GC xenograft growth. (A) Schematic diagram of the treatment model using SUMOylation and ATF6 inhibitors. (B) Representative images of xenograft tumors from the indicated groups in the CDX mouse model. Scale bar, 1 cm. (C) Tumor growth curves of the CDX mouse model measured at the indicated time points (*n* = 5 per group). (D) Tumor weights of excised xenografts from each group at the experimental endpoint. Data are presented as mean ± SD (*n* = 5). Statistical analysis was performed using unpaired 2-tailed Student’s *t* test. (E) IHC staining of circRAD23B-208aa, Ki67, PDIA5, TXNRD1, and HERPUD1 in xenograft tumor tissues from the indicated groups. Representative images are shown. Scale bar, 50 μm. (F) Graphical abstract. The circRAD23B-encoded protein circRAD23B-208aa stabilizes PDIA5 via UBC9-mediated SUMOylation, activates the ATF6 arm of the UPR, and promotes GC tumorigenesis and metastasis. Data presented as mean ± SD. *P < 0.05, **P < 0.01.

Moreover, we performed xenograft intervention experiments using pharmacological inhibitors in the context of circRAD23B-208aa knockdown. Mice bearing MKN-28 xenografts were divided into 5 groups: sh-NC control, sh-circRAD23B-208aa plus vehicle [dimethyl sulfoxide (DMSO)], sh-circRAD23B-208aa plus the SUMOylation inhibitor TAK-981, sh-circRAD23B-208aa plus the ATF6 inhibitor Ceapin-A7, and sh-circRAD23B-208aa plus both inhibitors. Tumor growth was monitored over time. Knockdown of circRAD23B-208aa alone markedly suppressed tumor growth compared to the control group. Notably, treatment with either TAK-981 or Ceapin-A7 further enhanced this inhibitory effect, and the combination of both inhibitors led to the most pronounced tumor suppression (Fig. 8A to C). Immunohistochemical staining of xenograft tissues revealed that expression of TXNRD1 and HERPUD1 was markedly reduced in the inhibitor-treated groups, correlating with the observed tumor inhibition (Fig. 8E). Importantly, body weight and serum levels of alanine aminotransferase, aspartate aminotransferase, blood urea nitrogen, and creatinine showed no significant differences between inhibitor-treated and control mice. Hematoxylin and eosin staining of major organs revealed no obvious histopathological abnormalities after inhibitor treatment (Fig. S8E to G). However, we acknowledge that neither TAK-981 nor Ceapin-A7 is specific to the circRAD23B-208aa/PDIA5 axis; the observed antitumor effects could involve other SUMOylation or ATF6 targets. Therefore, these results should be interpreted as a proof-of-concept that SUMOylation or ATF6 blockade can suppress tumors in which the circRAD23B-208aa/PDIA5 axis is active.

Collectively, these results demonstrate that circRAD23B-208aa stabilizes PDIA5 via UBC9-mediated SUMOylation, facilitating ATF6 dissociation, nuclear translocation, and transcriptional activation of TXNRD1 and HERPUD1. This pathway alleviates ER stress and promotes survival in GC cells (Fig. 8F).

## Discussion

The emerging role of translatable circRNAs has unveiled a new layer of gene regulation in cancer biology. CircRNA-derived proteins are increasingly recognized as key regulators of oncogenic signaling, metabolic reprogramming, and therapy resistance through diverse mechanisms such as protein stabilization, complex modulation, and epigenetic remodeling. Their frequent association with advanced disease stage and poor prognosis further underscores their potential as biomarkers and therapeutic targets. Within this context, our study identifies circRAD23B as a novel coding circRNA markedly up-regulated in GC. Through integrated ribosomal profiling and ORF prediction, we demonstrated its capacity to encode a 208-amino acid protein, circRAD23B-208aa. Critically, by developing a custom antibody targeting its sequence containing the unique back-splice junction, we provided definitive evidence of its endogenous expression in GC cells and clinical specimens—an essential advance beyond in silico predictions. Moreover, the association of circRAD23B-208aa expression with advanced tumor stage, metastasis, and inferior survival, together with ROC curve analyses, supports its potential prognostic value, while our functional evidence further identifies circRAD23B-208aa as a driver of gastric oncogenesis.

Beyond its expression pattern, circRAD23B-208aa exerts robust pro-tumorigenic functions. Notably, rescue experiments using start codon mutants or linearized RNA constructs confirmed that these oncogenic effects strictly depend on the protein product itself, rather than the parent circRNA acting as a molecular sponge or scaffold. Control experiments using IRES elements alone further excluded the possibility that the observed phenotypes arise from the IRES sequence functioning as a noncoding RNA. These findings firmly establish circRAD23B-208aa as a functionally independent oncogene, distinguishing it from many canonical circRNAs that operate primarily at the RNA level.

Mechanistically, we uncovered a novel mode of action through which circRAD23B-208aa exerts its effects. Using unbiased Co-IP coupled with mass spectrometry, we identified PDIA5—a disulfide isomerase central to ER stress response—as its interacting partner. This interaction was further validated in vivo by PLAs on xenograft tumor tissues, confirming its physiological relevance. Further investigation revealed that circRAD23B-208aa recruits the E2 conjugase UBC9 to catalyze SUMOylation of PDIA5 at lysine 25. This conclusion is supported by multiple lines of evidence: circRAD23B-208aa enhanced PDIA5-UBC9 association, the K25R mutation abolished PDIA5 SUMOylation and its stabilization by circRAD23B-208aa, and ubiquitination assays demonstrated that SUMOylation at this site antagonizes STUB1-mediated ubiquitination and subsequent proteasomal degradation.

SUMOylation, a dynamic posttranslational modification analogous to ubiquitination, regulates diverse cellular processes including protein stability, subcellular localization, and transcriptional activity [39–41]. For instance, in GC, the SUMO E3 ligase TRIM28 promotes PD-L1 stability and transcription to facilitate tumor immune evasion, while CBX4 modifies and stabilizes YAP1 to drive malignant progression [42,43]. Aberrant SUMOylation has been implicated in tumor progression, yet its regulation by noncanonical coding RNAs remains largely unexplored [44]. To our knowledge, this represents the first report of a circRNA-encoded protein directly engaging the SUMOylation machinery to stabilize a target protein. This SUMO modification competes with STUB1-mediated ubiquitination, shifting the equilibrium toward PDIA5 stabilization. This finding not only elucidates a new regulatory mechanism controlling PDIA5 turnover but also highlights a previously unrecognized crosstalk between circRNA-derived peptides and the SUMO–ubiquitin axis.

The stabilization of PDIA5 has profound implications for ER proteostasis and the UPR. The UPR is an adaptive signaling network that maintains ER homeostasis under stress conditions, and its dysregulation has been increasingly implicated in cancer progression, therapy resistance, and metabolic adaptation [45,46]. We demonstrated that elevated PDIA5 facilitates the reductive activation, Golgi processing, and nuclear translocation of ATF6, a major transducer of the UPR. This positions PDIA5—and by extension, circRAD23B-208aa—as an upstream regulator of a key UPR branch. Using catalytically inactive PDIA5 mutants, we further established that the enzymatic activity of PDIA5 is indispensable for its oncogenic functions downstream of circRAD23B-208aa. However, the exact molecular mechanism by which PDIA5 controls ATF6 maturation—including whether it directly remodels disulfide bonds in ATF6—remains to be determined in future biochemical studies. Notably, we confirmed that this regulation is specific to the ATF6 branch, as circRAD23B-208aa knockdown did not affect XBP1 splicing or the phosphorylation of IRE1 and eIF2α. Subsequent transcriptomic and chromatin analyses revealed that nuclear ATF6 drives the transcription of TXNRD1 and HERPUD1, critical effectors that enhance protein-folding capacity and ER-associated degradation, thereby alleviating ER stress and promoting cancer cell survival. This multi-tiered mechanism—linking a circRNA-encoded peptide to SUMOylation-dependent stabilization of PDIA5 and ultimately to ATF6-mediated UPR activation—provides a new perspective on how cancer cells exploit noncanonical translation products to maintain proteostatic balance under stress conditions.

The therapeutic implications of our findings are noteworthy. Using pharmacological inhibitors in xenograft models, we demonstrated that targeting SUMOylation with TAK-981 (a SUMOylation inhibitor) or Ceapin-A7 (an ATF6 inhibitor) markedly suppressed tumor growth, with combination therapy achieving the most pronounced effect. While a direct inhibitor targeting circRAD23B-208aa would offer superior specificity, developing such an agent presents considerable medicinal chemistry challenges given its nature as an intracellular protein functioning through protein–protein interactions. As structural information becomes available, future efforts could explore virtual screening or peptide-based approaches to target circRAD23B-208aa or its critical interaction interfaces.

Several limitations of our study should be acknowledged. A critical limitation is the exclusive use of immunodeficient mice in our in vivo experiments. Due to the lack of sequence homology between human circRAD23B and its mouse counterparts, we could not assess the impact of circRAD23B-208aa on tumor–immune interactions. Future studies using humanized mouse models or transgenic mice expressing human circRAD23B are warranted to address this question [47]. Additionally, our tail-vein experimental lung colonization assay bypasses the initial steps of local invasion and intravasation, and thus does not fully recapitulate spontaneous metastasis from an orthotopic primary tumor. Orthotopic gastric wall injection models, though technically demanding [48], would provide more clinically relevant validation and should be pursued in future studies. We also acknowledge that our custom antibody, while validated by knockdown and mass spectrometry, is not a commercially validated reagent with absolute specificity; however, it represents the best available tool for detecting endogenous circRAD23B-208aa at present.

In summary, our study substantially advances the field in several key aspects: It identifies circRAD23B-208aa as a novel oncogenic protein in GC; it delineates a unique mechanism of PDIA5 stabilization through SUMOylation mediated by UBC9 recruitment; and it defines a functional axis connecting circRNA translation output to adaptive ER stress response in GC. These findings underscore the importance of considering the coding potential of circRNAs in cancer biology and highlight the therapeutic potential of targeting the circRAD23B-208aa/PDIA5/ATF6 signaling node—particularly in tumors exhibiting heightened UPR activity.

## Materials and Methods

### Tissue samples

Tissue specimens were acquired from 60 patients diagnosed with GC at the First Affiliated Hospital of Nanjing Medical University between 2021 and 2023. All cases were histopathologically verified as primary gastric adenocarcinomas, and no patients had received radiotherapy or chemotherapy prior to surgery. Comprehensive follow-up data were available for all included cases. Immediately following resection, surgical specimens were flash-frozen in liquid nitrogen and subsequently stored at −80 °C under standardized biobanking protocols. The study protocol was approved by the Institutional Review Board of Nanjing Medical University and conducted in accordance with the ethical principles of the Declaration of Helsinki. Written informed consent was obtained from all participants. Patient demographic information, including gender, age, tumor size, TNM stage, survival, and cause of death, with the corresponding tissues and microarrays is presented in Table S1.

### Cell culture

GES-1 cells [American Type Culture Collection (ATCC), catalog no. CRL-14672] and HEK-293T cells (ATCC, catalog no. CRL-3216) were cultured in high-glucose Dulbecco’s modified Eagle’s medium (DMEM; Thermo Fisher Scientific, catalog no. 11965092) supplemented with 10% fetal bovine serum (FBS; Gibco, catalog no. 10270106) and 1% penicillin–streptomycin (PS; Beyotime, catalog no. C0222). The human GC cell lines MKN-28 (Cell Resource Center, Shanghai Institute of Life Sciences, catalog no. TCHu 99), HGC-27 (Cell Resource Center, Shanghai Institute of Life Sciences, catalog no. TCHu 101), and AGS (ATCC, catalog no. CRL-1739) were maintained in RPMI 1640 medium (Gibco, catalog no. 11875093) containing 10% FBS and 1% PS. All cells were incubated at 37 °C in a humidified atmosphere of 5% CO₂. Culture flasks and plates were γ-irradiated, and glassware was sterilized by autoclaving at 121 °C. All cell lines were routinely tested for mycoplasma contamination using the MycoAlert Kit (Lonza, catalog no. LT07-318) to confirm sterile conditions.

### RNA preparation and in vitro transcription

In vitro transcription for the generation of biotin-labeled RNA was carried out using a DNA template containing exon 5 of circRAD23B, whose sequence was obtained from the National Center for Biotechnology Information (NCBI) database. Forward and reverse primers containing the T7 promoter sequence (TAATACGACTCACTATAGGG) were designed to amplify the target region by PCR. The amplified products were separated on a 1% agarose gel to confirm the expected size, then purified and quantified. In vitro transcription was performed using biotin-labeled ribonucleotides in the reaction mixture, incubated at 37 °C for 4 h. To remove DNA template, the product was treated with deoxyribonuclease (DNase) I (1 U/μl) at 37 °C for 20 min. The transcribed RNA was purified by isopropanol precipitation and resuspended in nuclease-free water, and its integrity was verified by denaturing gel electrophoresis.

### RNase R digestion assay

Total RNA was extracted from HGC-27 and MKN-28 cells using TRIzol reagent (Invitrogen) and allocated to either experimental or control groups. To distinguish between circular and linear RNA forms, the experimental samples were incubated with RNase R (3 U/μg RNA; GenDepot, #R4001) in 1× reaction buffer containing 10 mM tris–HCl (pH 8.0), 100 mM KCl, and 0.1 mM MgCl₂ at 37 °C for 15 min. Control samples were processed identically but without enzyme. Reactions were terminated through extraction with acid-phenol:chloroform (pH 4.5), and RNA was recovered by ethanol precipitation. The integrity of RNA was assessed via 1.5% agarose gel electrophoresis. Subsequently, the relative levels of circRAD23B and linear RAD23B transcripts were determined by quantitative RT-PCR using glyceraldehyde-3-phosphate dehydrogenase (GAPDH) as an internal reference.

### RNA pull-down assays

RNA pull-down assays were performed using the Pierce Magnetic RNA-Protein Pull-Down Kit (Thermo Fisher Scientific, catalog no. 20164) to determine whether a specific fragment of circRAD23B pre-mRNA is capable of pulling down U2AF65. Cells were lysed in an ice-cold buffer provided in the kit, supplemented with protease inhibitors. The lysates were centrifuged at 13,000*g* for 15 min at 4 °C, and the supernatants were collected and treated with RNase inhibitors. Input samples (50 μl) were set aside for later analysis. The remaining lysates were incubated with streptavidin magnetic beads that had been pre-bound with biotin-labeled RNA probes corresponding to the target circRAD23B pre-mRNA sequence. The RNA-bound complexes were subsequently eluted and subjected to Western blot analysis.

### Proximity ligation assay

PLA was performed to detect protein–protein interactions in both cultured cells and formalin-fixed paraffin-embedded (FFPE) tumor tissues using the Duolink PLA Kit (Thermo Fisher Scientific, catalog no. DUO92101) according to the manufacturer’s instructions.

For cultured cells, samples were fixed with 4% paraformaldehyde for 15 min at room temperature, permeabilized with 0.5% Triton X-100 for 10 min, and blocked with 5% bovine serum albumin (BSA) for 1 h at room temperature. The interaction between PDIA5 and UBC9 was detected following the same experimental procedure.

For FFPE tissue sections from xenograft tumors, slides were deparaffinized in xylene, rehydrated through a graded ethanol series, and subjected to heat-induced antigen retrieval in citrate buffer (pH 6.0) at 95 °C for 20 min. Sections were then permeabilized with 0.5% Triton X-100 for 10 min and blocked with 5% BSA for 1 h at room temperature.

Both cell and tissue samples were incubated overnight at 4 °C with primary antibodies: rabbit anti-circRAD23B-208aa and mouse anti-PDIA5. After washing, samples were incubated with PLA probes (Duolink Anti-Rabbit PLUS and Anti-Mouse MINUS) for 1 h at room temperature, followed by ligation and amplification steps at 37 °C according to the manufacturer’s protocol. Fluorescent signals were visualized at excitation/emission = 594/624 nm using a confocal microscope (Zeiss LSM 880). Images were analyzed with ZEN software (Zeiss) and ImageJ. Negative controls included samples incubated with either primary antibody omitted or replaced with normal IgG.

### Insulin turbidity assay

Recombinant PDIA5-WT and PDIA5-CA (C53S/C56S) were expressed in HEK-293T cells and purified by anti-Flag affinity chromatography. Reactions were performed in 96-well plates containing 0.15 mM insulin, 1 mM dithiothreitol (DTT), and the indicated concentrations of PDIA5 proteins (0.5, 1.0, or 2.0 μM) in 100 mM potassium phosphate buffer (pH 7.0) with 0.2 mM EDTA. A no-enzyme control (DTT only) was included. Absorbance at 650 nm was measured every 1 min for 60 min at 25 °C using a microplate reader. Reductase activity (ΔA650/min) was calculated by linear regression from the linear phase (4 to 20 min) of each kinetic curve. Specific activity was normalized to protein concentration and expressed as units per mg of PDIA5. All experiments were performed 3 times independently, each with 3 technical replicates.

### PROTEOSTAT aggresome staining

PROTEOSTAT aggresome detection was performed in MKN-28 and HGC-27 GC cells using the PROTEOSTAT Aggresome Detection Kit (Enzo Life Sciences, catalog no. ENZ-51035-K100) according to the manufacturer’s instructions. Cells were seeded into 24-well plates containing sterile glass coverslips and treated according to experimental requirements. After treatment, cells were washed with phosphate-buffered saline (PBS), fixed with 4% paraformaldehyde for 15 min, and permeabilized with 0.5% Triton X-100 in PBS for 10 min. The cells were then incubated with the PROTEOSTAT dye (1:2,000 dilution in PBS) for 30 min at room temperature in the dark. After staining, cells were washed 3 times with PBS and mounted with antifade mounting medium containing 4′,6-diamidino-2-phenylindole (DAPI) (Beyotime, catalog no. P0131). Images were acquired using a laser scanning confocal microscope (Zeiss LSM 880) with appropriate excitation/emission settings for the dye (excitation ~550 nm, emission ~600 nm).

### ATP level measurement

Intracellular ATP levels were measured using a luciferase-based CellTiter-Glo Luminescent Cell Viability Assay kit (Promega, catalog no. G7570) according to the manufacturer’s instructions. GC cells (MKN-28 and HGC-27) were seeded in 96-well plates at a density of 5 × 10^3^ cells per well and cultured overnight. Cells were then transfected with control small interfering RNA (siRNA) (si-NC) or specific siRNAs targeting circRAD23B-208aa, PDIA5, TXNRD1, or HERPUD1. After 48 h of transfection, cells were equilibrated at room temperature for 30 min, and an equal volume of CellTiter-Glo reagent was added to each well. The plates were shaken for 2 min to induce cell lysis and then incubated at room temperature for 10 min to stabilize the luminescent signal. Luminescence was recorded using a multifunction microplate reader (BioTek Synergy H1). ATP levels were normalized to protein concentration measured using a bicinchoninic acid (BCA) assay (Beyotime, catalog no. P0012) and expressed as fold change relative to the si-NC control group.

### ERSE luciferase reporter assay

The ERSE luciferase reporter plasmid (ERSE-Luc) was constructed by inserting 3 tandem repeats of the ERSE core sequence (CCAATN₉CCACG) into the pGL4.26 vector (Promega) upstream of the minimal TATA box. For normalization, the pRL-TK plasmid (Promega), which constitutively expresses Renilla luciferase under the HSV-TK promoter, was cotransfected. MKN28 and HGC-27 GC cells were seeded in 96-well white plates at a density of 1.5 × 10^4^ cells per well and cultured overnight. Cells were cotransfected with 100 ng of ERSE-Luc firefly luciferase reporter plasmid and 10 ng of pRL-TK using Lipofectamine 3000 (Thermo Fisher Scientific) according to the manufacturer’s instructions. For knockdown or overexpression experiments, cells were additionally cotransfected with shRNA plasmids, siRNAs, or expression plasmids encoding PDIA5-WT, PDIA5-CA, or PDIA5-K25R as indicated. Tunicamycin (1 μg/ml) was used as a positive control for ER stress, and Ceapin-A7 (5 μM) was used as a specific ATF6 inhibitor. At 48 h post-transfection, cells were lysed with 1× Passive Lysis Buffer (Promega), and firefly and Renilla luciferase activities were measured sequentially using the Dual-Luciferase Reporter Assay System (Promega) on a GloMax 96 Microplate Luminometer (Promega). Firefly luminescence was normalized to Renilla luminescence to obtain relative luciferase activity.

### Chromatin immunoprecipitation

ChIP assays were conducted to examine the enrichment of the transcription factor ATF6 at the promoter regions of TXNRD1 and HERPUD1 in GC cell lines MKN-28 and HGC-27, using the SimpleChIP Plus Enzymatic Chromatin IP Kit (#9005, Cell Signaling Technology, Danvers, MA, USA) according to the manufacturer’s instructions. Briefly, approximately 1 × 10^7^ cells per sample were cross-linked with 1% formaldehyde for 10 min at room temperature, and the reaction was quenched with glycine. Cells were lysed, and chromatin was digested with micrococcal nuclease to generate DNA fragments ranging from 200 to 500 base pairs (bp). For each immunoprecipitation, 5 μg of chromatin was incubated overnight at 4 °C with rotation with an anti-ATF6 antibody (Cell Signaling Technology, catalog no. 65880; 1:50 dilution). Normal rabbit IgG (#2729, Cell Signaling Technology) was used as a negative control. Antibody–chromatin complexes were captured using ChIP-grade Protein G Magnetic Beads and washed under low- and high-salt conditions. After elution, cross-links were reversed, and DNA was purified. The precipitated DNA was analyzed by qRT-PCR to detect the presence of *TXNRD1* and *HERPUD1* promoter sequences. Results were normalized to Input DNA and expressed as percent Input.

### Mass spectrometry

Proteomic profiling was performed by Beijing Bio-Tech Pack Technology Co. Ltd. Proteins were extracted from GC patient tissues using radioimmunoprecipitation assay (RIPA) buffer supplemented with protease and phosphatase inhibitors, and concentrations were determined with a BCA assay. For cultured MKN-28 cells (5 × 10^6^), extraction was carried out with NP-40 lysis buffer. Proteins were separated via SDS-PAGE. Tissue samples (100 μg) underwent in-gel digestion with Trypsin Gold (37 °C, 16 h) and were subsequently desalted using C18 StageTips. Liquid chromatography–tandem mass spectrometry (LC-MS/MS) was conducted on a Q Exactive HF-X mass spectrometer operated in data-dependent acquisition (DDA) mode, coupled to a UPLC BEH C18 column with a 120-min acetonitrile gradient. Data were analyzed using MaxQuant (v2.1.3) against the UniProt Human proteome database (release 2023_01) with label-free quantification (LFQ). Proteins exhibiting |fold change| > 1.5 and *P* < 0.05 (Student’s *t* test) were defined as significantly altered.

For interaction analyses, immunoprecipitated complexes of circRAD23B-208aa-Flag or PDIA5 were examined by LC-MS/MS. Candidate interacting proteins were identified using Spectronaut 16 under a false discovery rate (FDR) ≤ 1%, requiring a minimum of 5-fold enrichment relative to immunoglobulin G (IgG) control (*P* < 0.01).

### RNA-seq

To identify differentially expressed circRNAs in GC, RNA-seq was performed using 5 pairs of matched GC and adjacent nontumor tissues. Total RNA was extracted with TRIzol reagent (Invitrogen) and treated with DNase I (RNase-free; Thermo Fisher) to remove gDNA. Ribosomal RNA was depleted using the Ribo-off rRNA Depletion Kit (Vazyme). RNA integrity was confirmed with an Agilent 2100 Bioanalyzer, and only samples with an RNA integrity number (RIN) ≥ 7.0 were used for sequencing. Strand-specific libraries were constructed using the VAHTSTM Stranded mRNA-seq Library Prep Kit (Vazyme) and sequenced on the Illumina NovaSeq 6000 platform with 150-bp paired-end reads. Raw reads were quality-controlled with FastQC and trimmed using Trimmomatic. Clean reads were aligned to the human reference genome (GRCh38) with STAR (v2.7.10a). circRNAs were detected and quantified using CIRI2 and CIRCexplorer3. Differential expression analysis was performed with DESeq2, applying thresholds of |log_2_(fold change) | > 1 and adjusted *P* value < 0.05. Selected circRNAs were validated by qRT-PCR using divergent primers.

### Ribosome nascent-chain complex sequencing

To identify translatable circRNAs in MKN-28 GC cells, RNC-seq was conducted. Cells were treated with cycloheximide (100 μg/ml) for 10 min to stall ribosomes, followed by lysis in polysome buffer. Lysates were centrifuged over a 10% to 50% sucrose gradient, and polysome-containing fractions were collected. RNA was extracted with TRIzol LS, treated with DNase I, and depleted of rRNA. Libraries were constructed using the VAHTS Stranded mRNA-seq Kit and sequenced on the Illumina NovaSeq 6000 platform with 150-bp paired-end reads. Reads were aligned to GRCh38 with STAR, and circRNAs were detected using CIRCexplorer3. Candidates with ≥2 back-splice reads in polysome fractions were considered putatively translated. ORF and IRES analyses were performed with IRESfinder and IRESite. Selected circRNAs were validated via qRT-PCR after polysome fractionation.

### Generation and validation of circRAD23B-208aa-specific polyclonal antibody

A custom polyclonal antibody was generated against a synthetic peptide that contains the unique back-splice junction sequence of circRAD23B-208aa (peptide sequence: “GGESTERED”, spanning the junction). The peptide was conjugated to keyhole limpet hemocyanin (KLH) for immunization. The peptide sequence was verified to be unique against all RAD23B isoforms and the human proteome by BLAST analysis. Rabbits received 4 booster immunizations at regular intervals with the antigen emulsified in Freund’s adjuvant. Antiserum titers were monitored by enzyme-linked immunosorbent assay (ELISA), yielding a final titer of ≥1:256,000. IgG was affinity-purified from the antiserum using Protein A/G chromatography, followed by antigen-specific purification, resulting in >90% purity as verified by SDS-PAGE. The antibody specifically recognized a single band of approximately 22 kDa in circRAD23B-208aa-overexpressing HEK-293T cell lysates via Western blot. Its specificity was further confirmed by immunofluorescence and immunohistochemistry, which showed selective staining of endogenous circRAD23B-208aa in GC cells and patient tissue sections.

### Animal experiments

All animal procedures were approved by the Institutional Animal Care and Use Committee of Nanjing Medical University (approval no. IACUC-2501018) and were conducted in compliance with institutional ethical guidelines for the care and use of laboratory animals.

#### Subcutaneous xenograft models

Male BALB/c nude mice (4 weeks old) were randomly assigned to experimental groups (*n* = 5 per group) for 3 independent subcutaneous tumor formation assays. In the first experiment, mice were injected with MKN-28 cells stably expressing control vector (Ctrl), circRAD23B-208aa knockdown (KD), KD plus circRAD23B-208aa overexpression (KD + circRAD23B OE), or KD plus translation-deficient circRAD23B mutant (KD + circRAD23B-ATG-mut). In the second experiment, mice were injected with MKN-28 cells stably expressing sh-NC, sh-circRAD23B-208aa reconstituted with empty vector (sh-circRAD23B-208aa + Vector), sh-circRAD23B-208aa reconstituted with wild-type PDIA5 (sh-circRAD23B-208aa + PDIA5 WT), or sh-circRAD23B-208aa reconstituted with the SUMOylation-deficient PDIA5 K25R mutant (sh-circRAD23B-208aa + PDIA5 K25R). For the third experiment, mice bearing sh-circRAD23B-208aa xenografts were treated with vehicle (DMSO), the SUMOylation inhibitor TAK-981 (subasumstat), the ATF6 inhibitor Ceapin-A7, or a combination of both inhibitors. TAK-981 (MedChemExpress, catalog no. HY-112686) was administered intravenously at 7.5 mg/kg twice weekly. Ceapin-A7 (Selleck Chemicals, catalog no. S8893) was prepared in 5% DMSO and 95% corn oil and administered intraperitoneally at 10 mg/kg every other day based on previous studies. Tumor growth was monitored every 3 days using a caliper, and tumor volume was calculated as (length × width^2^)/2. After 4 weeks, all mice were euthanized, and tumors were excised, weighed, and processed for further analysis.

#### Experimental lung metastasis model

For in vivo metastasis assays, HGC-27 cells stably expressing Ctrl, circRAD23B-208aa KD, KD + circRAD23B OE, or KD + circRAD23B ATG-mut were harvested and resuspended in PBS. Cells (2 × 10^5^ in 100 μl of PBS) were injected into the lateral tail vein of male BALB/c nude mice (4 weeks old, *n* = 5 per group). Six weeks after injection, all mice were euthanized, and lungs were harvested. The number of surface metastatic nodules was counted under a dissection microscope. For histological examination, lung tissues were fixed in 4% paraformaldehyde, embedded in paraffin, sectioned, and stained with H&E.

### Statistical analysis

Statistical analyses were conducted using GraphPad Prism (v9.0), SPSS (v26.0), or R (v4.2.0). Data are expressed as mean ± standard error of the mean (SEM) from at least 3 independent biological replicates. For comparisons between 2 groups, a 2-tailed Student’s *t* test was used. Comparisons among multiple groups were performed using one-way analysis of variance (ANOVA) or multi-factor ANOVA, as appropriate. If data did not meet assumptions of normality or homogeneity of variances, nonparametric tests were applied: the Mann-Whitney *U* test for 2-group comparisons and the Kruskal–Wallis test for multi-group comparisons. Correlation analyses were carried out using Pearson’s or Spearman’s correlation coefficients depending on data distribution characteristics. A *P* value less than 0.05 was considered statistically significant.

## Data Availability

The datasets supporting the findings of this study are included within the article. Requests for materials should be addressed to M.S.
