# Supplementary material for: CircRAD23B-208aa Promotes Gastric Cancer Progression by Activating the Unfolded Protein Response through PDIA5 SUMOylation
Source: Research (Wash D C). 2026 Jul 17;9:1357. doi: 10.34133/research.1357 (PMC13376384; doi:10.34133/research.1357)
Supplement: Supplementary 1 — Supplementary Methods Figs. S1 to S8 Tables S1 to S3 [file research.1357.f1.zip › Supplementary Information.docx]

Supplementary information for
**CircRAD23B-208aa Promotes Gastric Cancer Progression by Activating the Unfolded Protein Response through PDIA5 SUMOylation**

**Authors**：Yuli Chen^1,2#^, Jiahao Guo^1#^, Ziwei Li^1#^, Shaokun Yu^1^, Xiao Ke^1^, Qinnan Chen^1^, Hao Wu^3,4,5*^, Ming Sun^1*^, Fengqi Nie^6*^ and Xianghua Liu^7*^

**Affiliations:**^1^Department of Oncology, Suzhou Cancer Center Core Laboratory, Oncology Laboratory of Medical Science and Technology Innovation Center, The Affiliated Suzhou Hospital of Nanjing Medical University, Suzhou Municipal Hospital, Gusu School, Nanjing Medical University, Suzhou, Jiangsu Province, China.

^2^Department of Transfusion Medicine, Key Laboratory of Jiangxi Province for Transfusion Medicine, The First Affiliated Hospital, Jiangxi Medical College, Nanchang University, Nanchang, Jiangxi Province, China.

^3^Department of Oncology, The First Affiliated Hospital of Nanjing Medical University, Nanjing, Jiangsu Province, China.

^4^Gastric Cancer Center, The First Affiliated Hospital of Nanjing Medical University, Nanjing, Jiangsu Province, China.

^5^Institute for Gastric Cancer Research, Nanjing Medical University, Nanjing, Jiangsu China.

^6^Department of Oncology, Second Affiliated Hospital, Nanjing Medical University, Nanjing, Jiangsu Province, China.

^7^Department of Biochemistry and Molecular Biology, School of Basic Medical Sciences, Nanjing Medical University, Nanjing, Jiangsu Province, China.

**This Supplementary information file includes：**
Supplementary methods
Supplementary Figure legends: Supplementary Fig.1 to Supplementary Fig.6
Supplementary Table 1 to 3

**Supplementary methods**

**Lentivirus packaging and stable cell line construction**

HEK-293FT cells were cultured in DMEM supplemented with 10% FBS at 37 ℃ under 5% CO₂ and expanded to the logarithmic growth phase. Twenty-four hours before transfection, cells were seeded into 10-cm dishes at a density of 5×10⁶ cells per dish in 10 mL of medium to reach approximately 80% confluency. One hour prior to transfection, the medium was replaced with 4 mL of fresh complete medium. For lentivirus production, 10 μg of plasmid DNA-consisting of the expression vector, PSPAX2, and pMD2.G at a mass ratio of 4:3:2-was diluted in 500 μL of serum-free DMEM. PolyJet transfection reagent (SignaGen Laboratories), used at twice the total plasmid mass, was added to the DNA mixture, incubated for 15 minutes at room temperature, and applied dropwise to the cells. After 12 hours, the medium was replaced. Viral supernatants were collected 48 hours post-transfection, filtered through 0.45 μm PVDF membranes, aliquoted, and stored at -80℃. To generate stable cell lines, HGC-27 and MKN-28 cells were infected with concentrated lentivirus at 30-50% confluency in the presence of 8 μg/mL polybrene. After 48 hours, puromycin (1 μg/mL) was added to select transduced cells. The selection medium was refreshed every day until all cells in the uninfected control group were completely eliminated. Puromycin-resistant pools were expanded and evaluated for target gene expression via Western blot analysis.

**Transient transfection and protein extraction**

For transient transfection, cells were seeded in six-well plates 24 hours in advance to reach 60-80% confluency. For siRNA transfection, Lipofectamine 2000 (Invitrogen) was diluted in 250 µL of serum-free Opti-MEM (Tube A), and 100-200 pmol of siRNA was separately diluted in 250 µL of the same medium (Tube B). After incubating both tubes for 5 minutes at room temperature, the solutions were combined, gently mixed, and incubated for 20 minutes in the dark to allow complex formation. Following two washes with pre-warmed PBS, the transfection complexes were added dropwise to the cells.

For protein extraction, transfected cells were washed twice with cold PBS and lysed on ice using RIPA buffer (Beyotime, P0013B) supplemented with protease inhibitors (Roche, 4693159001). The lysates were centrifuged at 12,000 × g for 15 minutes at 4℃. Supernatants were collected, and protein concentration was determined using a BCA Protein Assay Kit (Thermo Scientific, 23225). Protein samples were mixed with 5 × SDS loading buffer, denatured at 100℃ for 10 minutes, and stored at -20℃ until further Western blot analysis.

**Cell Counting Kit-8 (CCK-8) Assay**

Cell proliferation was assessed using the CCK-8 assay (Dojindo, Japan). MKN-28 and HGC-27 cells were seeded into 96-well plates at a density of 1×10³ cells per well in 100 µL of complete medium. After incubation for 0, 24, 48, 72, and 96 hours, 10 µL of CCK-8 reagent was added to each well and incubated at 37℃ for 2 hours. The absorbance was measured at 450 nm using a microplate reader (BioTek, USA). Each experiment was performed with six replicate wells and repeated in three independent biological replicates.
**Colony Formation Assay**

For the colony formation assay, MKN-28 and HGC-27 cells were seeded in 6-well plates at a density of 1000 cells per well and cultured for 9-14 days. The medium was replaced every three days. Once visible colonies had formed, cells were fixed with 4% paraformaldehyde for 30 minutes, washed gently with PBS, and stained with 0.1% crystal violet for 15 minutes at room temperature. After washing with distilled water to remove residual dye, the plates were air-dried. Colonies containing more than 50 cells were counted under a microscope, and images were captured. Quantitative analysis was performed using ImageJ software (National Institutes of Health, USA).

**Cell migration and invasion assays**

Cell migration was evaluated using Transwell chambers (8 μm pore size; Corning). Briefly, 5×10⁴ cells in 200 μL of serum-free medium were seeded into the upper chamber. The lower chamber was filled with 600 μL of complete medium containing 10% FBS as a chemoattractant. After 24 hours of incubation at 37℃, non-migratory cells on the upper surface were carefully removed with a cotton swab. Migrated cells on the lower membrane were fixed with 4% paraformaldehyde for 20 minutes, stained with 0.1% crystal violet for 15 minutes, and gently washed with PBS. The membranes were air-dried, and images of five random fields per well were captured under an inverted microscope. Quantification was performed using ImageJ software.

For the invasion assay, Transwell inserts were pre-coated with Matrigel (Corning, #356234) diluted 1:8 in ice-cold serum-free DMEM and polymerized at 37℃ for 2 hours. Then, 1×10⁵ cells in 200 μL serum-free medium were plated in the upper chamber. The lower chamber contained 600 μL complete medium with 10% FBS. After 36–48 hours of culture, non-invaded cells were removed from the upper side, and invaded cells on the lower surface were fixed, stained, and quantified following the same procedure as described for the migration assay.

**Subcellular fractionation**

Nuclear and cytoplasmic RNA fractions were isolated using a modified sucrose gradient-based separation protocol. Cells were lysed in 0.25% trypsin and centrifuged at 1,000 × g for 10 minutes at 4℃. The pellet was resuspended in 400 µL of lysis buffer containing 10% NP-40, incubated on ice for 5 minutes, and mixed with 1 mL of ice-cold sucrose gradient buffer (20% sucrose, 10 mM HEPES, pH 7.4). After centrifugation at 4,000 × g for 10 minutes at 4℃, the cytoplasmic supernatant was carefully collected. The nuclear pellet was washed with 0.5 M EDTA-PBS and recentrifuged. RNA from both fractions was extracted using TRIzol LS reagent (Invitrogen) according to the manufacturer’s instructions. Reverse transcription and qRT-PCR were performed to detect RNA distribution, with GAPDH and U6 serving as cytoplasmic and nuclear controls, respectively.

For protein subcellular localization studies, nuclear and cytoplasmic fractions were prepared using a commercial kit (NE-PER™ Nuclear and Cytoplasmic Extraction Reagents, Thermo Scientific, #78833) according to the manufacturer’s protocol. Briefly, cells were harvested and lysed in cytoplasmic extraction reagent supplemented with protease and phosphatase inhibitors. The lysate was centrifuged at 12,000 × g for 10 minutes at 4℃ to obtain the cytoplasmic fraction. The insoluble pellet was resuspended in nuclear extraction reagent and vortexed vigorously, followed by centrifugation at 12,000 × g for 10 minutes to collect the nuclear fraction. Protein concentrations were determined using a BCA assay. Fraction purity was confirmed by Western blot using anti-GAPDH (cytoplasmic marker) and anti-Histone H3 (nuclear marker) antibodies.

**Plasmid construction and mutagenesis**

The circRAD23B overexpression plasmid was constructed by cloning the circularizable flanking sequences of RAD23B into a PLVX-Puro-ciR backbone (YouBio, China) using EcoRI and BamHI restriction sites. For site-directed mutagenesis (e.g., circRAD23B-mut), mutations were introduced using Phanta® Max Super-Fidelity DNA Polymerase (Vazyme, P505-d1) according to the manufacturer’s instructions. Each 50 µL reaction contained 50 ng of methylated template, 0.4 µM of each primer, and 1 U/µL polymerase. The PCR program consisted of an initial denaturation at 95℃ for 30 s; 30 cycles of denaturation at 95℃ for 15 s, annealing at 60–65℃ for 15 s, and extension at 72℃ for 1 min/kb; followed by a final extension at 72℃ for 5 min. The amplified product was treated with DpnI (Thermo Scientific, ER1701) to digest the methylated template, purified, re-circularized using Exnase II (Vazyme, C302-03), and transformed into DH5α competent cells. All mutant constructs were verified by Sanger sequencing (Tsingke Biotechnology).

**Actinomycin D assay**

MKN-28 and HGC-27 cells were treated with 2.5 μg/mL actinomycin D (MedChemExpress, cat. no. HY-17559) to inhibit transcription and harvested at 0, 3, 6, 12, and 24 hours post-treatment. Total RNA was extracted at each time point using TRIzol reagent (Invitrogen) and reverse-transcribed into cDNA with PrimeScript RT Master Mix (Takara, cat. no. RR036A). The expression levels of circRAD23B and linear RAD23B were quantified by qRT-PCR using TB Green Premix Ex Taq II (Takara, cat. no. RR820A). GAPDH served as an internal control for normalization. The half-life of each transcript was calculated based on the relative RNA levels remaining across the time points.

**RNA immunoprecipitation (RIP) assays**
RIP assays were performed to investigate RNA-protein interactions. Protein A/G magnetic beads were pre-washed with ice-cold PBS containing 0.1% Tween-20. The beads were then incubated with 5 μg of anti-U2AF65 antibody (Abcam, cat. no. ab37530) or normal IgG control (Cell Signaling Technology, cat. no. 3900S) overnight at 4℃ with gentle rotation. Subsequently, pre-cleared cell lysates were added to the antibody-bound beads and incubated for 4 hours at 4℃. After incubation, the beads were washed sequentially with buffers of increasing stringency: first with a low-salt buffer (0.1% SDS, 1% NP-40, 150 mM NaCl), followed by a high-salt buffer (0.1% SDS, 1% NP-40, 300 mM NaCl), and finally with a standard wash buffer. RNA bound to the immunocomplexes was extracted using TRIzol LS reagent (Invitrogen), purified by ethanol precipitation, and analyzed by quantitative RT-PCR.

# Dual-luciferase reporter assay To investigate the effect of ATF6 on the transcriptional activity of TXNRD1 and HERPUD1, dual-luciferase reporter assays were performed. Promoter sequences of TXNRD1 and HERPUD1 were cloned into the pGL3-Basic firefly luciferase reporter vector (Promega). HGC-27 and MKN-28 cells were co-transfected with the recombinant reporter plasmids and an ATF6 overexpression plasmid or empty vector control using Hieff Trans® Liposomal Transfection Reagent (Yeasen, cat. no. 40802ES03). Forty-eight hours after transfection, firefly and Renilla luciferase activities were measured with the Dual-Luciferase® Reporter Assay System (Promega, cat. no. E1910) on a GloMax® Navigator luminometer (Promega). The firefly luciferase activity was normalized to that of Renilla luciferase for each sample.

**Immunofluorescence (IF)**

MKN-28 and HGC-27 cells were plated into confocal dishes pre-coated with poly-L-lysine (0.1 mg/mL) and cultured until 60-70% confluency. Cells were washed three times with cold PBS and fixed with 4% paraformaldehyde for 20 minutes at room temperature. After permeabilization with 0.3% Triton X-100 in PBS for 10 minutes, cells were blocked with 5% bovine serum albumin (BSA) for 1 hour at room temperature. Primary antibodies were applied and incubated overnight at 4℃. The following day, cells were incubated with species-matched secondary antibodies-Alexa Fluor® 488-conjugated goat anti-mouse (1:500; Abcam, ab150113) or Cy3-conjugated goat anti-rabbit IgG (1:500; Abcam, ab97075)-for 2 hours at room temperature in the dark. Nuclei were counterstained with DAPI (1 μg/mL; Sigma-Aldrich, D9542) for 5 minutes. Finally, samples were mounted with anti-fade mounting medium (Invitrogen, P36965) and visualized using a laser-scanning confocal microscope (Zeiss LSM 880).

**Thioflavin T (ThT) Staining**

ThT staining was carried out to detect amyloid-like protein aggregates in MKN-28 and HGC-27 cells. Cells were cultured on coverslips in 24-well plates and treated as indicated. After treatment, cells were fixed with 4% PFA for 15 minutes, permeabilized with 0.1% Triton X-100 for 10 minutes, and then incubated with 0.05% Thioflavin T (Sigma-Aldrich, cat. no. T3516) in PBS for 20 minutes at room temperature in the dark. Following incubation, cells were washed three times with PBS and mounted using antifade mounting medium with DAPI. Fluorescence images were obtained with a confocal microscope (Zeiss LSM 880) using an excitation wavelength of 440 nm and an emission range of 480-520 nm.

***In vitro* SUMOylation assay**

Recombinant circRAD23B-208aa protein was expressed in HEK293T cells with a Flag tag and purified by anti‑Flag affinity chromatography (Sigma‑Aldrich, cat. no. M8823). PDIA5-WT and PDIA5-K25R were expressed and purified similarly. UBC9 (SUMO E2) and SAE1/SAE2 (SUMO E1 heterodimer) were purchased from Boston Biochem (cat. no. E-305) and SUMO‑2 was purchased from R&D Systems (cat. no. UL-601). The SUMOylation reaction was performed in a 50 μL volume containing 50 mM Tris‑HCl (pH 7.5), 10 mM MgCl₂, 1 mM DTT, 5 mM ATP, 0.5 μg of SAE1/SAE2 (E1), 0.5 μg of UBC9 (E2), 2 μg of SUMO‑2, and 1 μg of PDIA5-WT or PDIA5-K25R as substrate. Purified circRAD23B-208aa was added at indicated concentrations (0.5, 1.0, 2.0 μg) to test its scaffold activity. RanBP2 fragment (Boston Biochem, cat. no. E-310) was used as a positive control for SUMO E3 ligase activity. Reactions were incubated at 30℃ for 90 minutes and terminated by adding 5× SDS loading buffer followed by boiling at 95℃ for 5 minutes. Samples were separated by 12% SDS‑PAGE and subjected to western blotting with anti‑SUMO2/3 antibody to detect SUMOylated PDIA5. To confirm specificity, control reactions were performed without UBC9, without SUMO‑2, or without ATP. All experiments were performed three times independently. Densitometric quantification was performed using ImageJ.

**Immunohistochemistry (IHC)**

Formalin-fixed, paraffin-embedded (FFPE) tissue sections (4 μm thick) from 70 paired gastric cancer and adjacent non-tumor samples were deparaffinized in xylene and rehydrated through a graded ethanol series. Antigen retrieval was performed by heating the sections in citrate buffer (pH 6.0) at 95 ℃ for 15 minutes. Endogenous peroxidase activity was quenched with 3% hydrogen peroxide for 15 minutes. After blocking with 10% normal goat serum for 1 hour at room temperature, sections were incubated overnight at 4℃ with a primary antibody against PDIA5. The following day, slides were incubated with a horseradish peroxidase (HRP)-conjugated secondary antibody for 1 hour at room temperature. Immunoreactivity was visualized using a DAB substrate kit (ZSGB-BIO, ZLI-9018), and nuclei were counterstained with hematoxylin. Sections were dehydrated, cleared in xylene, and mounted with neutral balsam. For the xenograft tumor tissues derived from nude mice, immunohistochemical detection of circRAD23B-208aa and Ki67 was performed using the same protocol with specific antibodies. All stained sections were evaluated by two independent pathologists.

**Supplementary Fig.1 to Supplementary Fig.8**

**Supplementary Fig 1. A,** Volcano plot of differentially expressed circRNAs between gastric cancer and adjacent normal tissues. **B,** QRT-PCR analysis of circRAD23B, circDYRK1A, circMTDH, circUBQLN1, and circCPSF6 in 5 paired gastric cancer and adjacent normal tissues. **C,** circRAD23B expression levels in gastric cancer cell lines by qRT-PCR. **D,** RNase R digestion assay for circRAD23B stability validation. **E,** Actinomycin D assay determining circRAD23B half-life. **F,** Schematic illustration of circRAD23B knockdown and overexpression constructs. **G,** qRT-PCR validation of circRAD23B knockdown and overexpression efficiency in the indicated gastric cancer cells. **H-I,** Cell proliferation assessed by CCK-8 (**H**) and colony formation assays (**I**) post-circRAD23B knockdown or overexpression. **J,** Transwell assays evaluating cell migration and invasion after circRAD23B knockdown. Scale bar，100 μm. Data presented as mean ± SD; * P<0.05，** P<0.01，*** P<0.001.

**Supplementary Fig 2. A,** Kaplan-Meier survival analysis of overall survival (OS), progression-free survival (PFS), and post-progression survival (PPS) in GC patients stratified by U2AF65 expression levels using the KM Plotter database. **B，**QRT-PCR validation of U2AF65 knockdown efficiency in GC cells. **C-D,** CCK-8 (**C**) and colony formation (**D**) assays assessing the proliferation of GC cells after U2AF65 knockdown. **E,** Transwell migration and invasion assays in gastric cancer cells after U2AF65 knockdown. Scale bar,100 μm. **F,** Densitometric quantification of the Western blot bands shown in Figure 2F. **G-H,** Schematic representation of IRES truncation constructs (G) and western blot validation of their expression efficiency (H). **I****,** Dual-luciferase reporter assays detecting IRES activity of the indicated constructs. **J,** Western Blot verifies the expression of circRAD23B-208aa in 48 paired gastric cancer and adjacent normal tissues. Data presented as mean±SD; * P<0.05，** P<0.01，*** P<0.001，ns, not significant.

**Supplementary Fig 3. A-B,** CCK-8 (**A**) and colony formation (**B**) assays assessing the proliferation of GC cells overexpressing the indicated IRES truncation constructs. **C,** Transwell migration and invasion assays in GC cells overexpressing the indicated IRES truncation constructs. Scale bar,100 μm. Data presented as mean±SD; ns, not significant.

**Supplementary Fig 4. A,** Co-IP assays detecting the interaction between circRAD23B-208aa and 10 candidate target proteins. **B,** Secondary spectrum of PDI5. **C,** Schematic diagram of the interaction between circRAD23B-208aa and PDIA5. **D,** PLA detecting the direct interaction between circRAD23B-208aa and PDIA5 in xenograft tumor tissues from the CDX mouse model in Figure 3E. **E,** Schematics of circRAD23B and PDIA5 truncation constructs. **F,** Western blot identifying interaction domains. **G,** QRT-PCR and Western blot analysis of PDIA5 expression after circRAD23B-208aa knockdown. **H,** PDIA5 knockdown efficiency verification by qRT-PCR. **I,** QRT-PCR and Western blot analysis of circRAD23B-208aa expression after PDIA5 knockdown. **J,** Two-step Co-IP verifying tripartite complex of circRAD23B-208aa, PDIA5, and UBC9. **K,** IF shows circRAD23B-208aa co-localization with PDIA5.Scale bar,20 μm. **L,** 3D binding model of the circRAD23B-208aa-PDIA5-UBC9 complex predicted by AlphaFold Server. Protein sequences were retrieved from UniProt and submitted for automated protein‑protein docking. circRAD23B-208aa is shown in blue, PDIA5 in pink, and UBC9 in green. Yellow dashed lines indicate hydrogen bond interactions. **M，**Co‑IP analysis of PDIA5 SUMOylation in subcutaneous xenograft tumors (Figure 3E) following circRAD23B‑208aa knockdown. **N,** UBC9 knockdown efficiency verification by western blot. **O,** IHC detection of PDIA5 expression levels in 30 tumor patient tissues of GC and 10 adjacent normal tissues. **P,** Western blot of PDIA5 expression in 12 paired gastric cancer tissues. **Q,** Correlation between PDIA5 and circRAD23B-208aa levels in 58 samples. Scale bar,100 μm. Data presented as mean±SD; * P<0.05, ** P<0.01, *** P<0.001, **** P<0.0001, ns, not significant.

**Supplementary Fig 5.** **A,** STUB1 knockdown efficiency verification by Western Blot. **B-D,** CCK-8(**B**), colony formation(**C**) and Transwell assays (**D**) showing impaired proliferation, migration, and invasion after PDIA5 knockdown (n=5; two-way ANOVA). Scale bar,100 μm. **E-F,** PROTEOSTAT (**E**) and Thioflavin T (ThT, **F**) staining detecting unfolded protein accumulation post-PDIA5 knockdown. Scale bar, 100 μm. **G,** Western blot analysis of eIF2α and BiP/GRP78 expression after PDIA5 knockdown. **H,** Intracellular ATP levels in GC cells after PDIA5 knockdown. ATP was measured using a luminescence-based assay and normalized to protein concentration. **I,** TEM showing ER dilation (left) and quantification of expanded ER (right) post-PDIA5 knockdown. Scale bar,20 μm. Data presented as mean±SD; * P<0.05, ** P<0.01, **** P<0.0001.

**Supplementary Fig 6. A,** KEGG enrichment analysis. **B,** Enrichment analysis shows UPR signaling pathway may be positively correlated with ER. **C,** Co-IP assay evaluating the effect of circRAD23B-208aa knockdown on the redox status of ATF6. **D,** QRT‑PCR analysis of IRE1 (XBP1s, EDEM1, Sec61a1) and PERK (ATF4, CHOP, ASNS, CHAC1) pathway target genes after circRAD23B‑208aa knockdown, with Tunicamycin as positive control. **E,** Western blot analysis of p-IRE1, p-eIF2α, and ATF4 expression in GC cells after circRAD23B-208aa knockdown. **F-G,** PROTEOSTAT (**F**) and Thioflavin T (**G**) staining detecting unfolded protein accumulation after circRAD23B-208aa knockdown. Scale bar,100 μm. **H,** Western blot analysis of eIF2α and BiP/GRP78 expression after circRAD23B-208aa knockdown. **I,** Intracellular ATP levels in GC cells after circRAD23B-208aa knockdown. ATP was measured using a luminescence-based assay and normalized to protein concentration. **J,** TEM showing ER dilation (left) and quantification of expanded ER (right) post-circRAD23B-208aa knockdown.Scale bar,20 μm. Data presented as mean±SD; * P<0.05, ** P<0.01, *** P<0.001, ns, not significant.

**Supplementary Fig 7. A,** Western blot validation of PDIA5 knockout (KO) and rescue efficiency in GC cells. PDIA5 KO cells were re-transfected with empty vector, wild-type PDIA5-HA (PDIA5-HA), or the catalytically inactive PDIA5-CA-Mut-HA. **B,** Cell proliferation assays in the indicated MKN-28 (left) and HGC-27 (right) cell lines measured by CCK-8 assays at the indicated time points. Statistical analysis was performed using two-way ANOVA. **C,** Colony formation assays in the indicated MKN-28 (left) and HGC-27 (right) cell lines. Representative images (top) and quantification of colony numbers (bottom) are shown. **D,** Transwell migration and invasion assays in the indicated MKN-28 and HGC-27 cell lines. Representative images (top) and quantification of migrated or invaded cells per field (bottom) are shown. Scale bar, 100 μm. **E,** TXNRD1 knockdown efficiency verification by western blot and qRT-PCR. **F-H,** CCK-8(**F**), colony formation(**G**) and Transwell assays (**H**) showing impaired proliferation, migration, and invasion after TXNRD1 knockdown (n=3; two-way ANOVA). Scale bar,100 μm. **I,** HERPUD1 knockdown efficiency verification by Western Blot and qRT-PCR. **J-L,** CCK-8(**J**), colony formation(**K**) and Transwell assays(**L**) showing impaired proliferation, migration, and invasion after HERPUD1 knockdown (n=3; two-way ANOVA). Scale bar,100 μm. Data presented as mean ± SD; * P<0.05，** P<0.01，*** P<0.001.

**Supplementary Fig 8. A-B,** PROTEOSTAT (**A**) and Thioflavin T (**B**) staining detecting unfolded protein accumulation after TXNRD1 and HERPUD1 knockdown. Scale bar,100 μm. **C,** Western blot analysis of eIF2α and BiP/GRP78 expression after TXNRD1 and HERPUD1 knockdown. **D,** Intracellular ATP levels in GC cells after TXNRD1 and HERPUD1 knockdown. ATP was measured using a luminescence-based assay and normalized to protein concentration. **E,** Body weight curves of mice bearing subcutaneous xenografts (Figure 8A). **F,** Serum ALT, AST, BUN, and Cr levels in each treatment group for toxicity evaluation. **G,** H&E staining of heart, liver, spleen, lung, and kidney in each treatment group for organ toxicity assessment. Data presented as mean ± SD; * P<0.05，** P<0.01，*** P<0.001.

**Supplementary Table 1: Correlation of the expression of circRAD23B-208aa with clinicopathologic features in gastric cancer.**

| Variables | n | circRAD23B-208aa expression | | P |
| --- | --- | --- | --- | --- |
|  |  | Low | High |  |
| **Gender** |  |  |  | 0.909 |
| Male | 42 | 18 | 24 |  |
| Female | 18 | 8 | 10 |  |
| **Age(years)** |  |  |  | 0.129 |
| ＜55 | 21 | 14 | 7 |  |
| ≥55 | 39 | 18 | 21 |  |
| **Tumor size(cm)** |  |  |  |  |
| ＜5 | 28 | 19 | 9 | 0.004** |
| ≥5 | 32 | 9 | 23 |  |
| **N stage** |  |  |  | 5.81e-6** |
| N0 | 25 | 18 | 7 |  |
| N1-2 | 35 | 5 | 30 |  |
| **TNM stage** |  |  |  | 6.28e-7** |
| I+II | 21 | 17 | 4 |  |
| III+IV | 39 | 6 | 33 |  |
| **Tumor differentiation** |  |  |  | 0.548 |
| High | 39 | 18 | 21 |  |
| Low | 21 | 8 | 13 |  |

Fold change (FC) (tumor tissues relative to normal tissues) is greater than or equal to 2.0 for high expression, and less than 2.0 for low expression. *P<0.05,**P＜0.01 was considered significant.

**Supplementary Table 2: Primer sequences for qRT-PCR**

| Gene | Forward | Reverse |
| --- | --- | --- |
| GAPDH | GTCAAGGCTGAGAACGGGAA | AAATGAGCCCCAGCCTTCTCA |
| U6 | CTCGCTTCGGCAGCACA | AACGCTTCACGAATTTGCGT |
| circRAD23B-Divergent | CAAGTTCTGGAGGTGAAAGC | GAGCAGTATCATCATTGAGG |
| circRAD23B-Convergent | GATGAGAAAAACTTTGTGGTGGGG | AAACTGGCTCTCAGGGCTGCAATT |
| SLC3A2 | CTGGTGCCGTGGTCATAATC | GCTCAGGTAATCGAGACGCC |
| CCN2 | GAAATGCTGCGAGGAGTG | CCCACAGGTCTTGGAACA |
| CBX4 | TCCAGAACAGGGAACGGCAGT | AACGACGGGCAAAGGTAGGCC |
| TRIB3 | CCATTTGGTCCTGACGGAAAGTCGGTCG | AGCCTTGAAGTCACAAGCCGTTTC |
| XBP1 | GAGTCCGCAGCAGGTG | GTGTCAGAGTCCATGGGA |
| DNAJB9 | CAGAGAGATTGCAGAAGCATATGA | GCTTCTTGGATCGAGTGTTTT |
| CPEB4 | ACAGTGACTTTGTGATGGATGG | TTATCATCGCAAGCTCCACA |
| HERPUD1 | TGGATTGGACCTATTCAGCAGC | GCAGGTACATAACAACGGTGGC |
| KLF4 | GGGAGAAGACACTGCGTCA | GGAAGCACTGGGGGAAGT |
| TXNRD1 | CGAGAACTATTGGCTTAGAGA | ACCGATGGCGTAGATGTA |
| U2AF65 | CGAGTGTGGGAGCCAAGAAT | CATACTCCTCGTCGTCCAGC |
| PDIA5 | GGACATTGTGGAGTGGCTGA | CGTGGAACATGACGAGGACA |
| RAD23B | TGTGGTGAGCTAGCGGATTC | GGTGGGGGCTTGTCACATT |
| ATF6 | CAGCAGGAACTCAGGGAGTG | AATGTGTCTCCCCTTCTGCG |

**Supplementary Table 3: siRNA/shRNA sequences**

|  | Gene | Sequence |
| --- | --- | --- |
| siRNA | U2AF65-1 5’-3’ | UUGAAGAAAUCCAUCAUGGCC |
|  |  | CCAUGAUGGAUUUCUUCAACG |
|  | U2AF65-2 5’-3’ | AUUCUUGUCCUGGUUAAUCUG |
|  |  | GAUUAACCAGGACAAGAAUUU |
|  | UBC9-1 5’-3’ | CAAGAAAUAAGGCGGAAUATT |
|  |  | UAUUCCGCCUUAUUUCUUGTT |
|  | UBC9-2 5’-3’ | GUCCAUGGAUUUACAGAAATT |
|  |  | UUUCUGUAAAUCCAUGGACTT |
|  | UBC9-3 5’-3’ | GGUUAAAUUUCCAGCCGGATT |
|  |  | UCCGGCUGGAAAUUUAACCTT |
|  | ATF6-1 5’-3’ | GUGAGCUACAAGUGUAUUATT |
|  |  | UAAUACACUUGUAGCUCACTT |
|  | ATF6-2 5’-3’ | CAAGAAGAAUGACAAAUAATT |
|  |  | UUAUUUGUCAUUCUUCUUGTT |
|  | PDIA5-1 5’-3’ | CCAUGAAAGACUAGGGAAATT |
|  |  | UUUCCCUAGUCUUUCAUGGTT |
|  | PDIA5-2 5’-3’ | GGAAGAUCCUGGAGCCAAATT |
|  |  | UUUGGCUCCAGGAUCUUCCTT |
|  | PDIA5-3 5’-3’ | GCAAGAAGAUGAAAGUUGATT |
|  |  | UCAACUUUCAUCUUCUUGCTT |
|  | STUB1-1 5’-3’ | GGAGATGGAGAGCTATGAT |
|  | STUB1-2 5’-3’ | GGAGCAGGGCAATCGTCTG |
|  | STUB1-3 5’-3’ | CTGTGAAGGCGCACTTCTT |
|  | TXNRD1-1 5’-3’ | GCGAUAUAUUGGAGGAUAATT |
|  |  | UUAUCCUCCAAUAUAUCGCTT |
|  | TXNRD1-2 5’-3’ | GAUAGAAGCUGUACAGAAUTT |
|  |  | AUUCUGUACAGCUUCUAUCTT |
|  | TXNRD1-3 5’-3’ | GGAAAUCAUUGAAGGAGAATT |
|  |  | UUCUCCUUCAAUGAUUUCCTT |
|  | HERPUD1-1 5’-3’ | GGAAGAAGAUGAUGAAAUATT |
|  |  | UAUUUCAUCAUCUUCUUCCTT |
|  | HERPUD1-2 5’-3’ | GCUGUUGUUGGAUCACCAATT |
|  |  | UUGGUGAUCCAACAACAGCTT |
|  | HERPUD1-3 5’-3’ | GGCUUGUCUUCAAGACUUUTT |
|  |  | AAAGUCUUGAAGACAAGCCTT |
|  | si-NC | UUCUCCGAACGUGUCACGUTT |
|  |  | ACGUGACACGUUCGGAGAATT |
| shRNA | si-h-hsa_circ_0087856_001 | ACAAGTTCTGGAGGTGAAA |
|  | si-h-hsa_circ_0087856_002 | AGTTCTGGAGGTGAAAGCA |
|  | si-h-hsa_circ_0087856_003 | CTGGAGGTGAAAGCACTGA |
