# Supplementary figures and images for: CircRAD23B-208aa Promotes Gastric Cancer Progression by Activating the Unfolded Protein Response through PDIA5 SUMOylation
Source: Research (Wash D C). 2026 Jul 17;9:1357. doi: 10.34133/research.1357 (PMC13376384; doi:10.34133/research.1357)

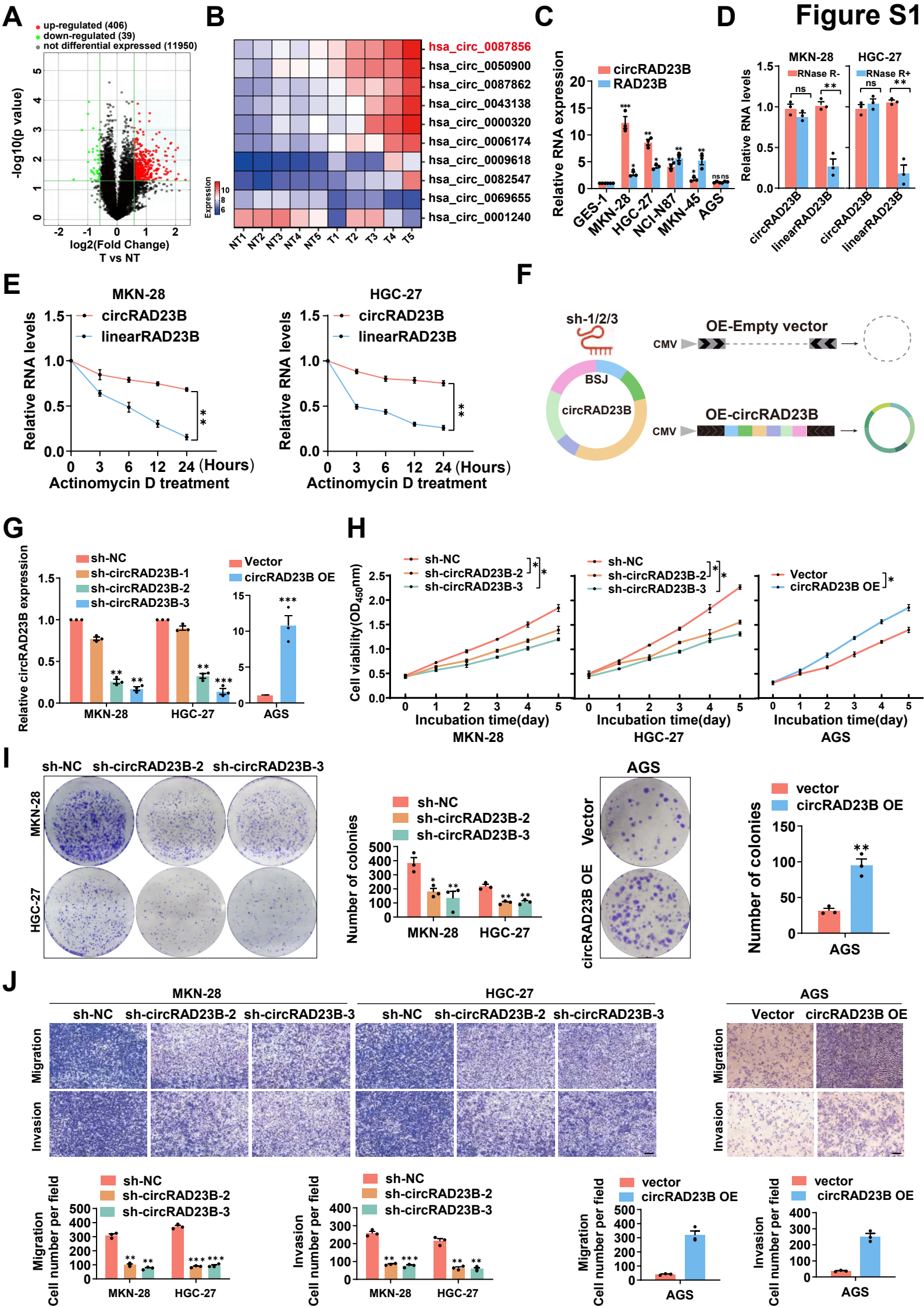

Supplement: Supplementary 1 — Supplementary Methods Figs. S1 to S8 Tables S1 to S3 [file research.1357.f1.zip › Supplementary Fig-1.pdf]

Figure S2

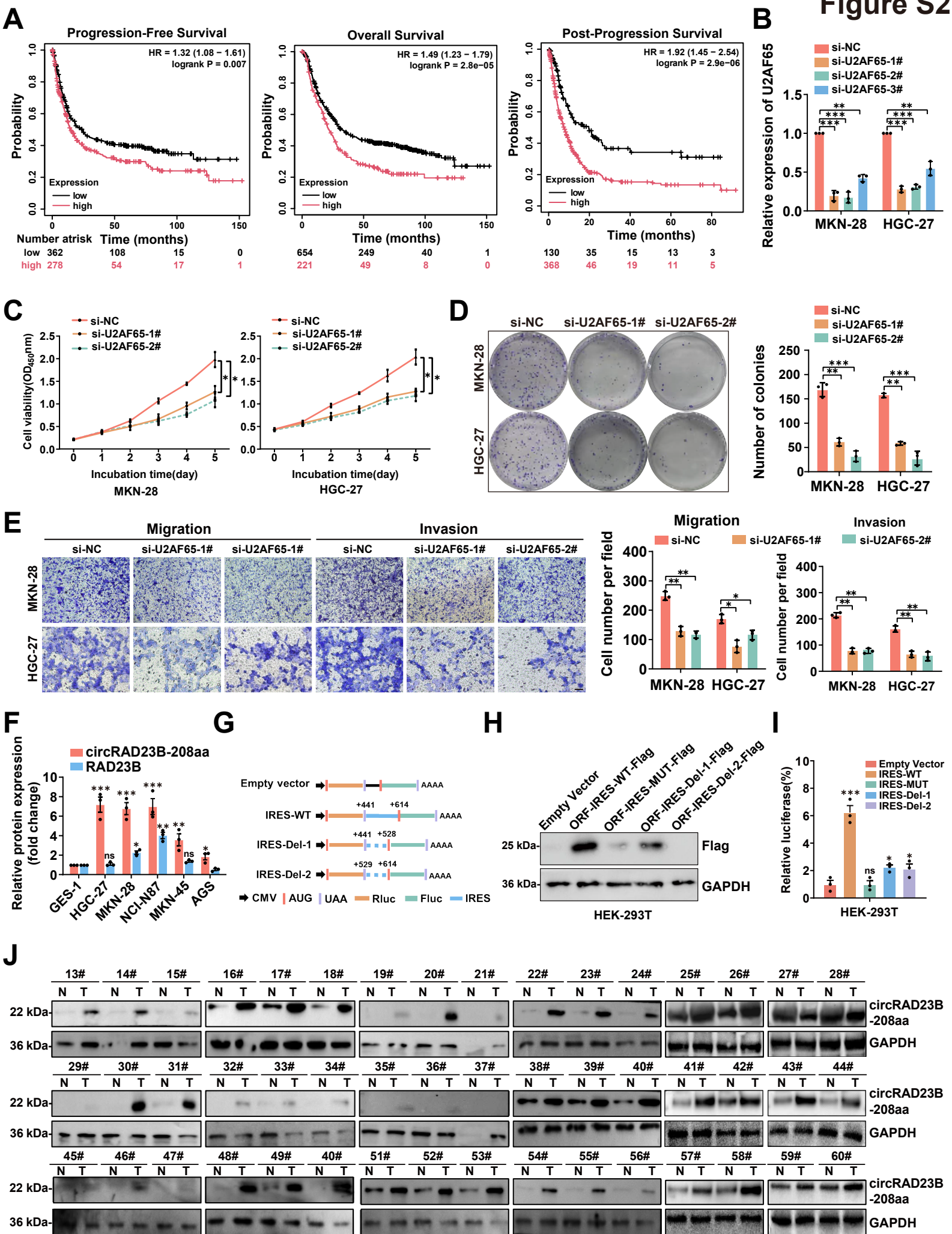

Supplement: Supplementary 1 — Supplementary Methods Figs. S1 to S8 Tables S1 to S3 [file research.1357.f1.zip › Supplementary Fig-2.pdf]

# Figure S3

## A

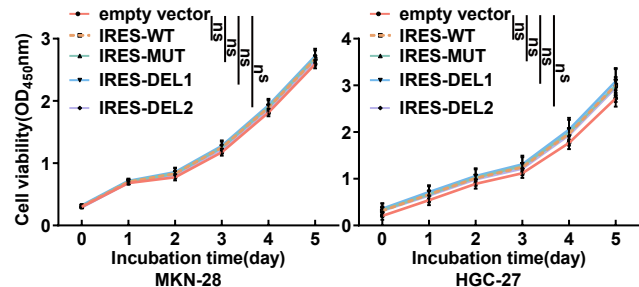

## B

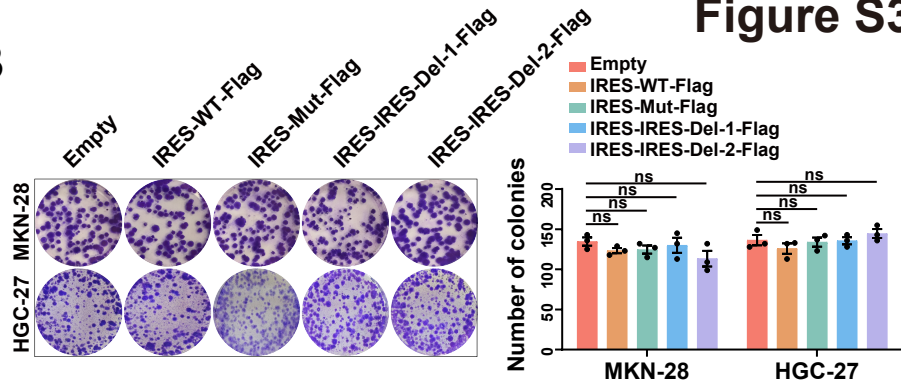

## C

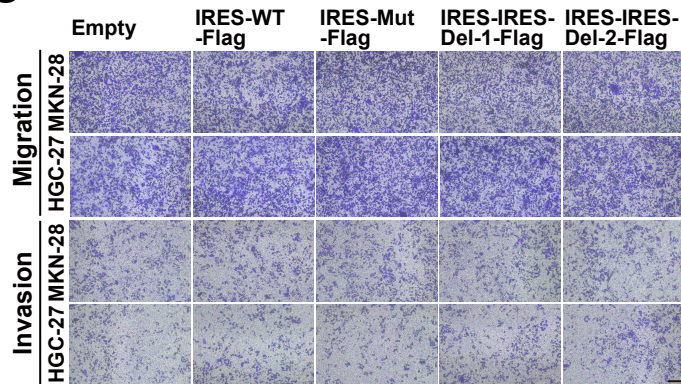

### Migration

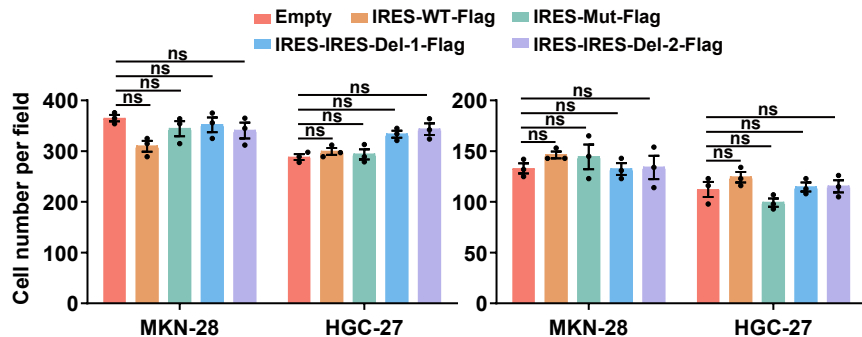

Supplement: Supplementary 1 — Supplementary Methods Figs. S1 to S8 Tables S1 to S3 [file research.1357.f1.zip › Supplementary Fig-3.pdf]

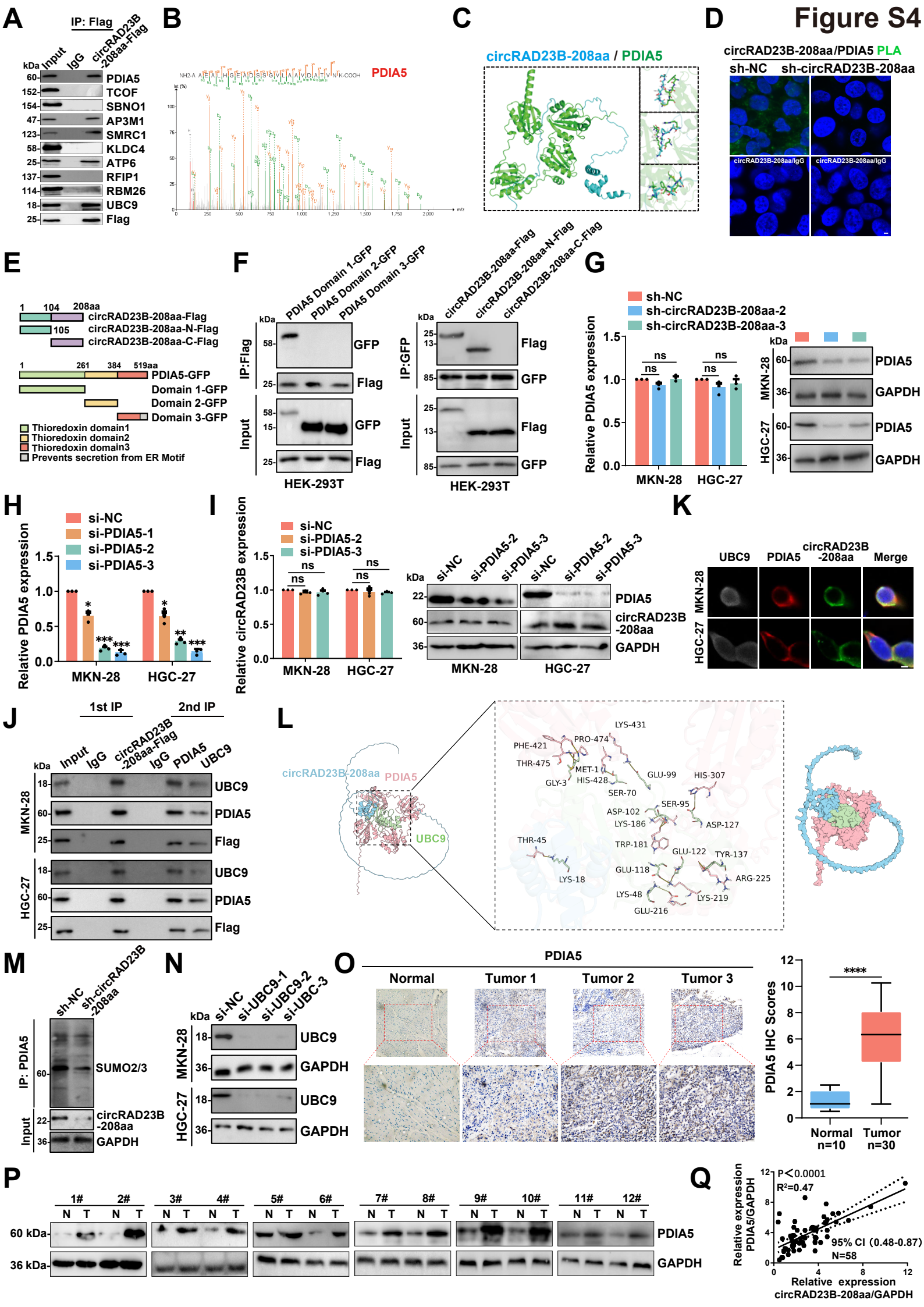

Supplement: Supplementary 1 — Supplementary Methods Figs. S1 to S8 Tables S1 to S3 [file research.1357.f1.zip › Supplementary Fig-4.pdf]

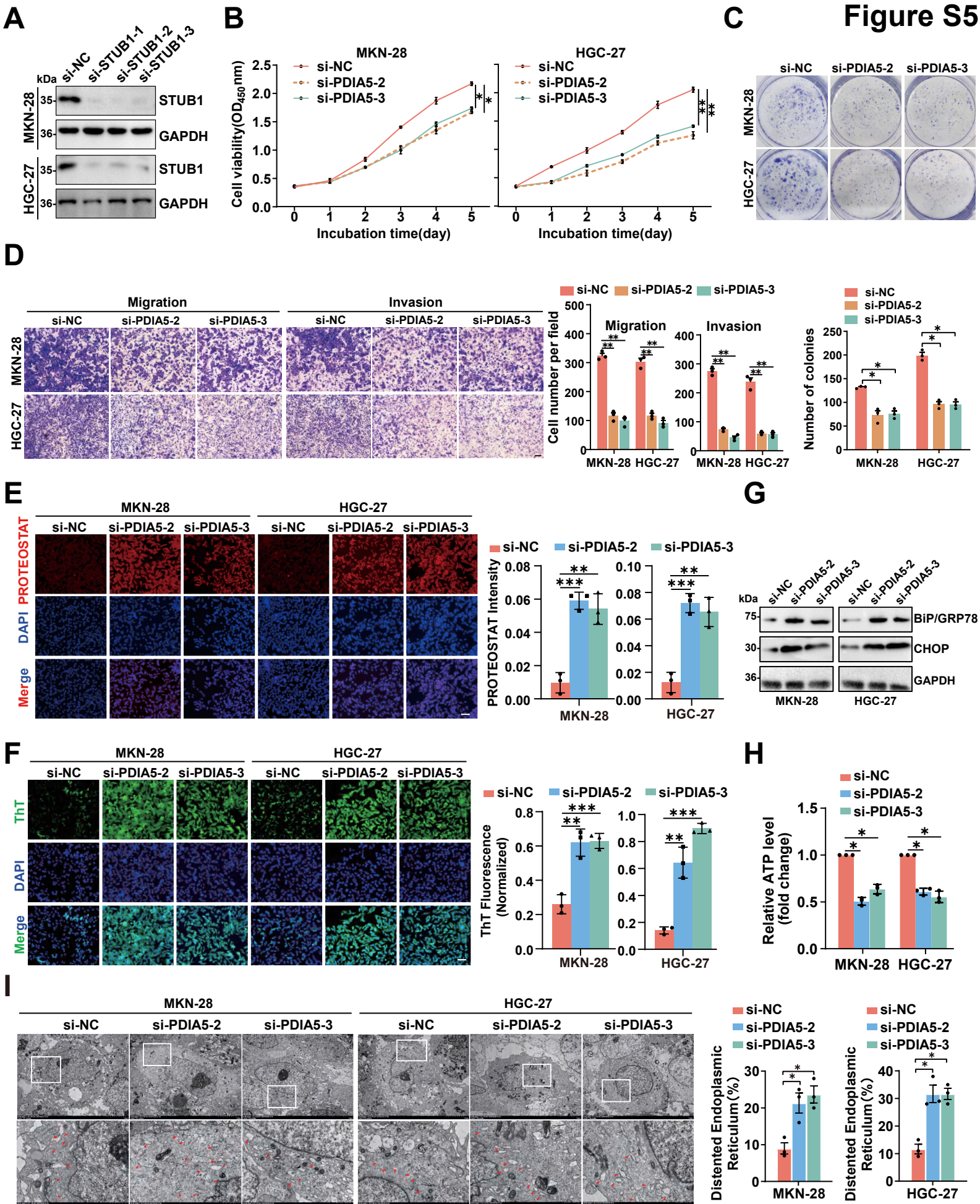

Supplement: Supplementary 1 — Supplementary Methods Figs. S1 to S8 Tables S1 to S3 [file research.1357.f1.zip › Supplementary Fig-5.pdf]

**A**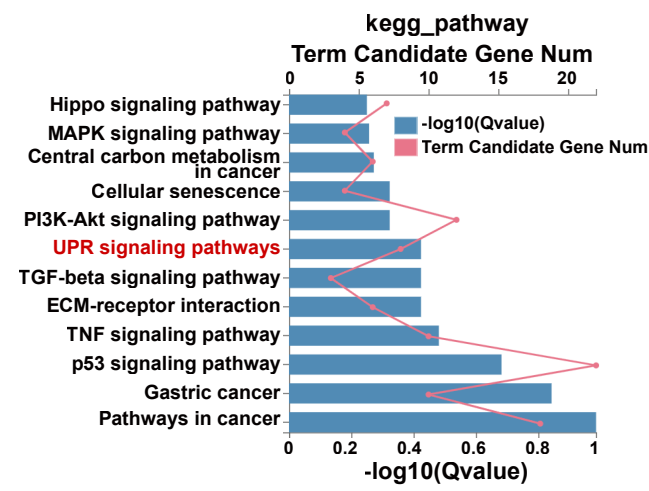**B**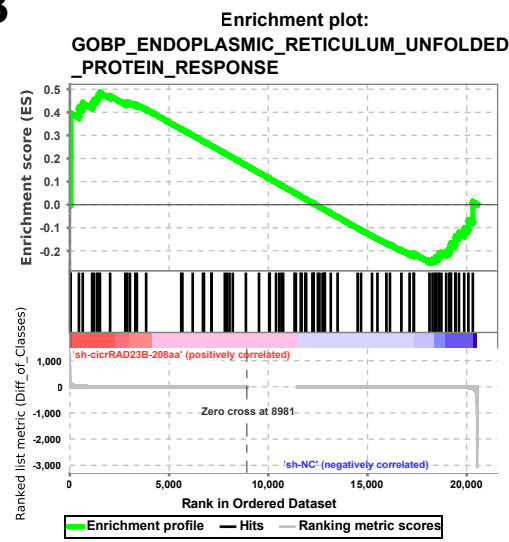**C**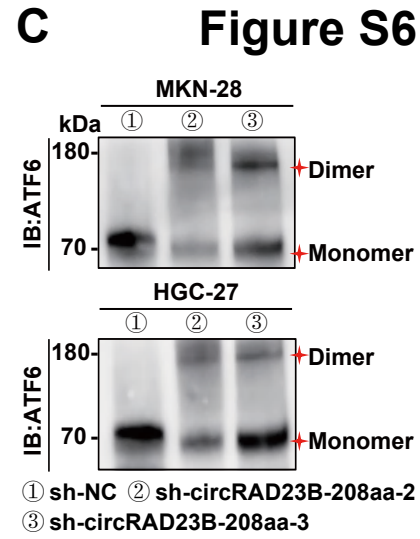**D**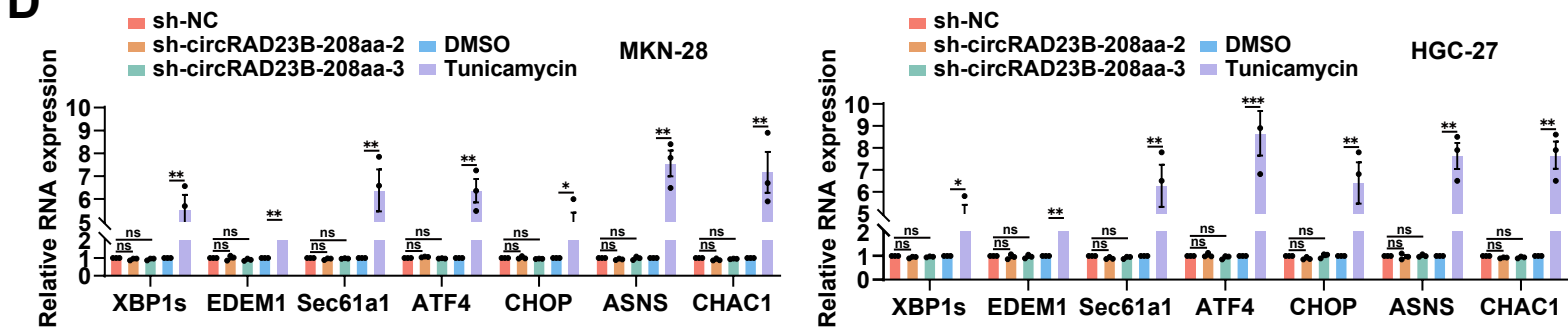**E**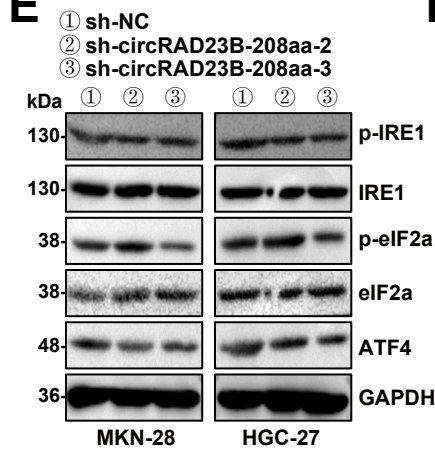**F**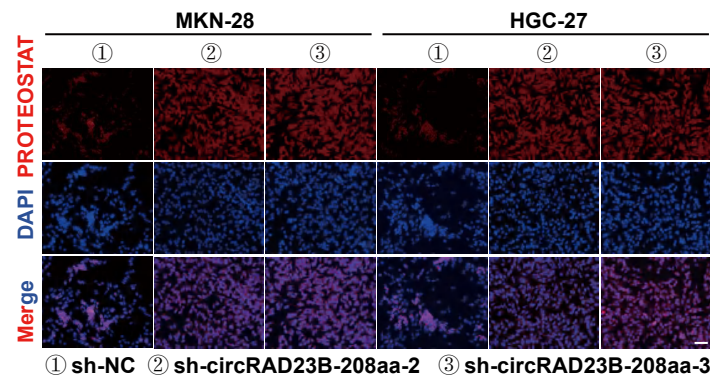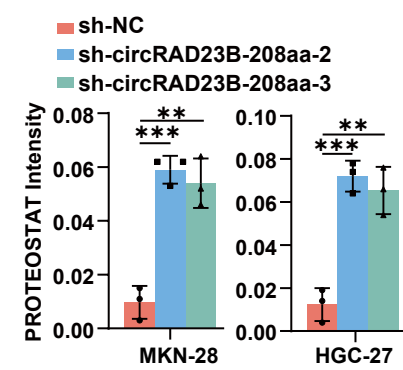**G**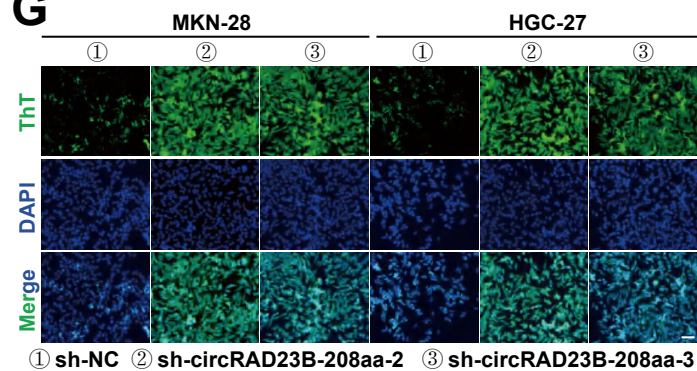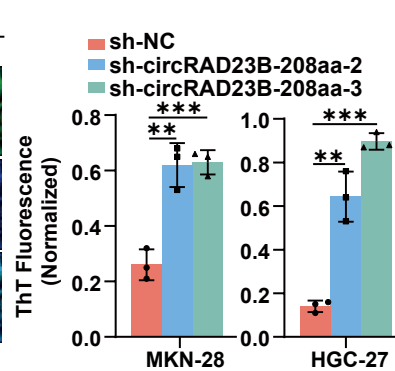**H**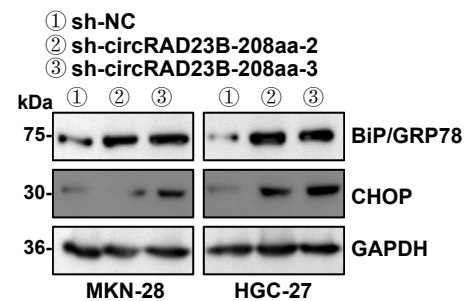**I**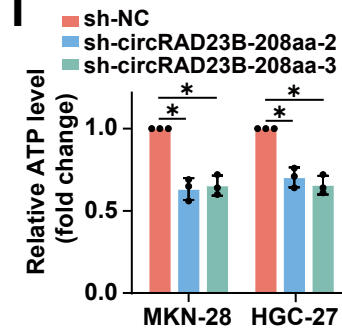**J**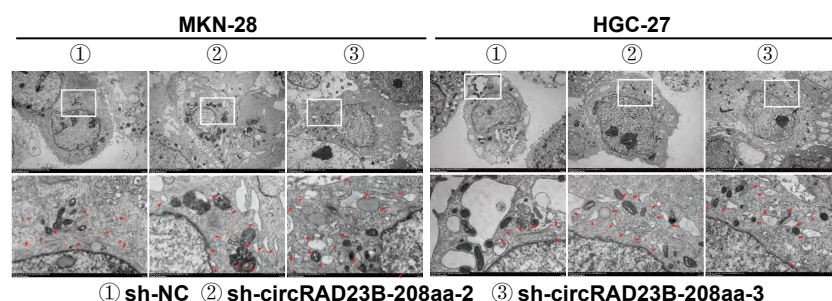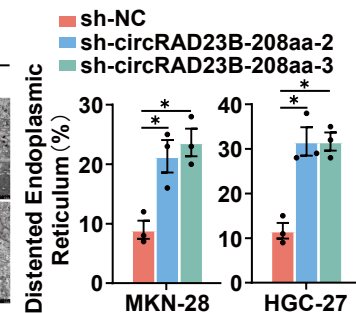

Supplement: Supplementary 1 — Supplementary Methods Figs. S1 to S8 Tables S1 to S3 [file research.1357.f1.zip › Supplementary Fig-6.pdf]

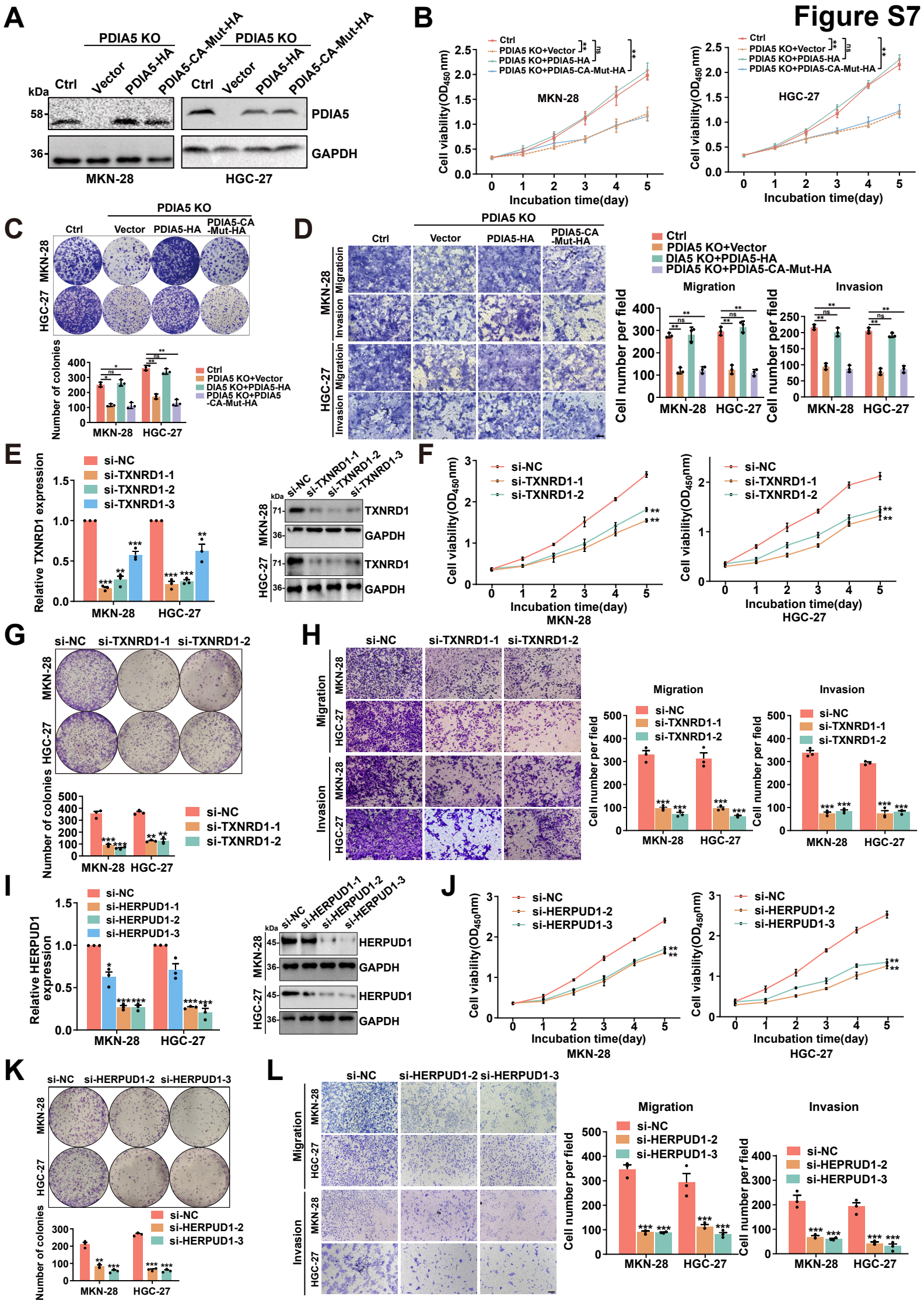

Supplement: Supplementary 1 — Supplementary Methods Figs. S1 to S8 Tables S1 to S3 [file research.1357.f1.zip › Supplementary Fig-7.pdf]

Figure S8

A

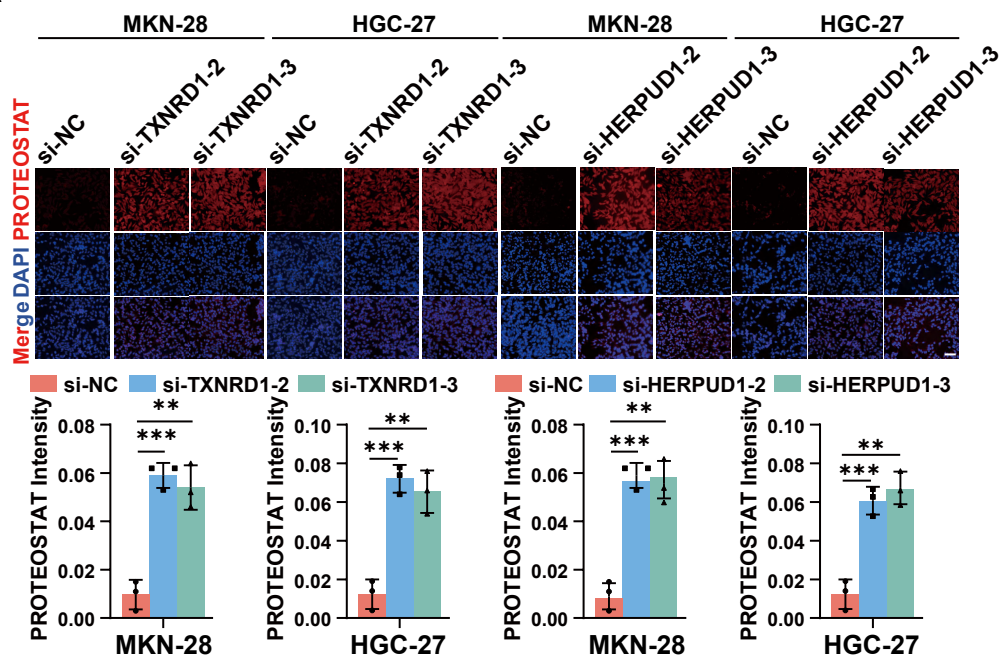

C

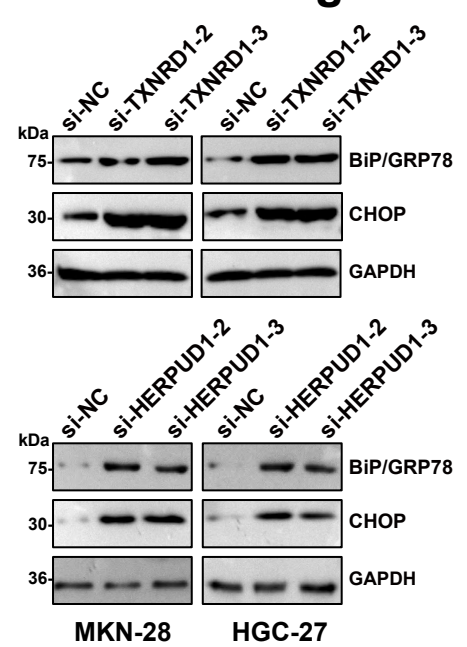

B

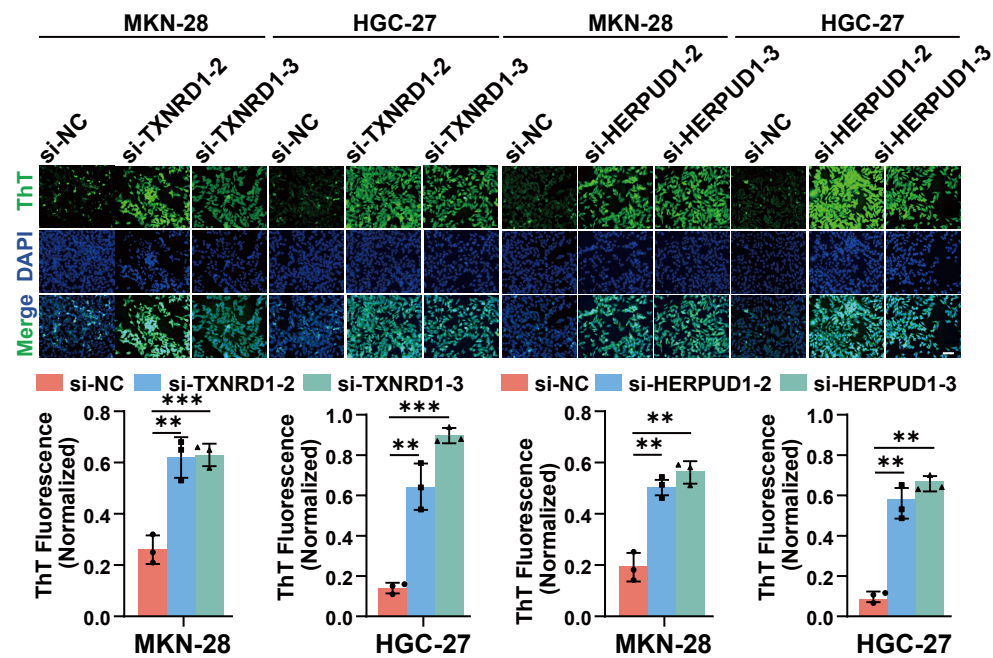

D

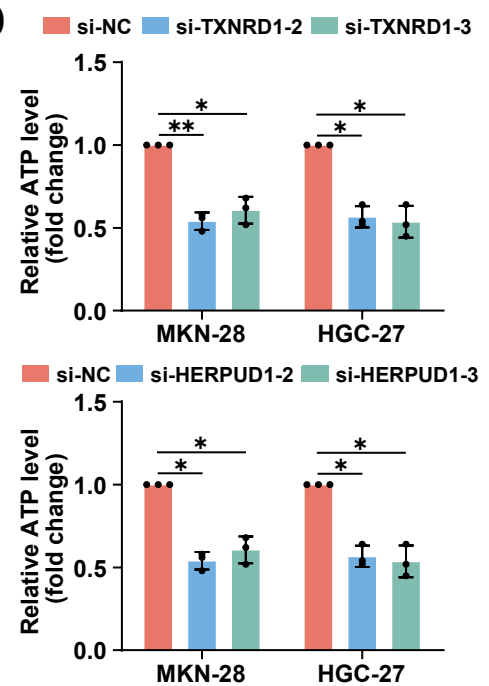

E

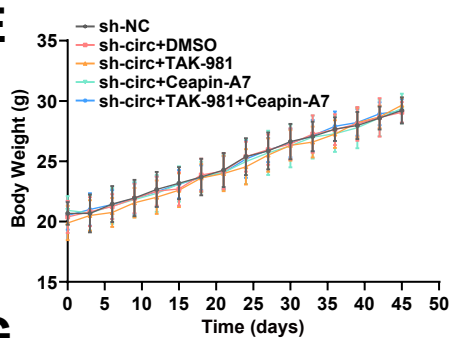

F

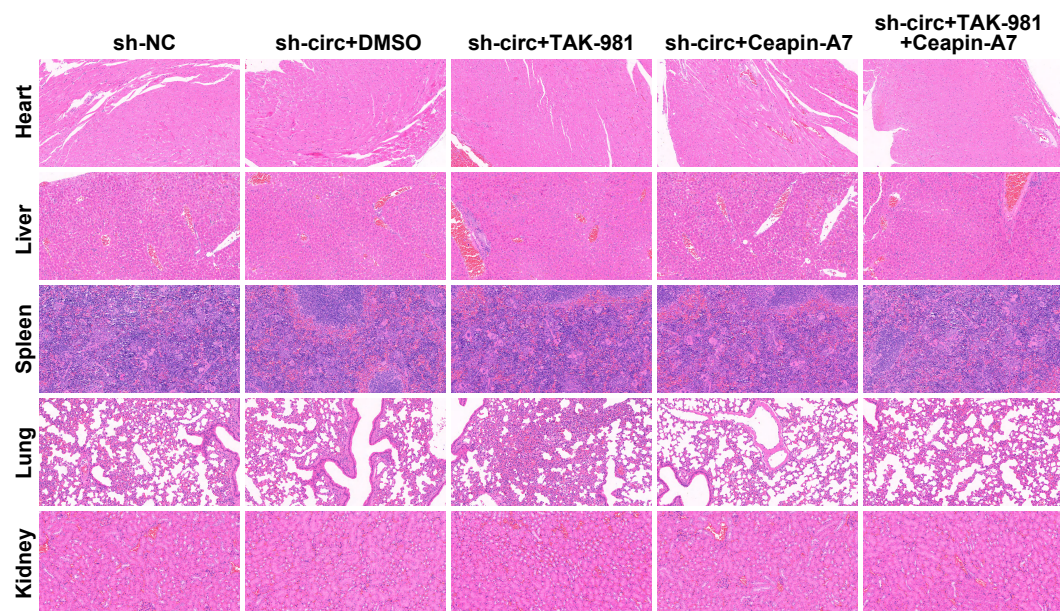

G

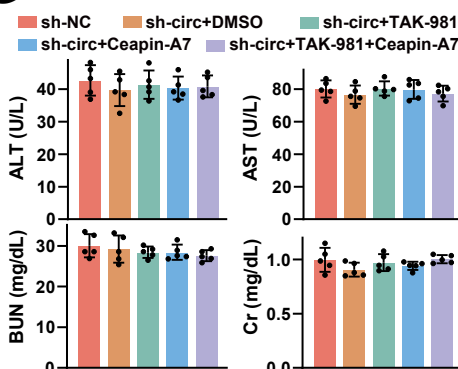

Supplement: Supplementary 1 — Supplementary Methods Figs. S1 to S8 Tables S1 to S3 [file research.1357.f1.zip › Supplementary Fig-8.pdf]
